# Supplementary material for: Dietary Approaches to the Management Of type 2 Diabetes (DIAMOND): protocol for a randomised feasibility trial
Source: BMJ Open. 2019 Jan 17;9(1):e026460. doi: 10.1136/bmjopen-2018-026460 (PMC6340425; doi:10.1136/bmjopen-2018-026460)
Supplement: Author's manuscript [file bmjopen-2018-026460.draft_revisions.pdf]

BMJ Open is committed to open peer review. As part of this commitment we make the peer review history of every article we publish publicly available.

When an article is published we post the peer reviewers' comments and the authors' responses online. We also post the versions of the paper that were used during peer review. These are the versions that the peer review comments apply to.

The versions of the paper that follow are the versions that were submitted during the peer review process. They are not the versions of record or the final published versions. They should not be cited or distributed as the published version of this manuscript.

BMJ Open is an open access journal and the full, final, typeset and author-corrected version of record of the manuscript is available on our site with no access controls, subscription charges or pay-per-view fees (http://bmjopen.bmj.com).

If you have any questions on BMJ Open's open peer review process please email info.bmjopen@bmj.com

BMJ Open

Dietary approaches to the management of type 2 diabetes (DIAMOND): protocol for a randomised feasibility trial

Journal:	*BMJ Open*
Manuscript ID	bmjopen-2018-026460
Article Type:	Protocol
Date Submitted by the Author:	03-Sep-2018
Complete List of Authors:	Morris, Elizabeth; University of Oxford, Nuffield Department of Primary Care Health Sciences; NIHR Biomedical Research Centre, Oxford<br>Aveyard, Paul; University of Oxford, Primary Care Health Sciences; NIHR Biomedical Research Centre, Oxford<br>Dyson, Pamela; University of Oxford , Oxford Centre for Diabetes, Endocrinology and Metabolism; NIHR Biomedical Research Centre, Oxford<br>Noreik, Michaela; University of Oxford Department of Primary Care Health Sciences; NIHR Biomedical Research Centre, Oxford<br>Bailey, Clare; Burnham Health Centre<br>Fox, Robin; Bicester Health Centre<br>Hoffman, Kathy; NHS Chiltern Clinical Commissioning Group<br>Tan, Garry; Oxford University Hospitals NHS Foundation Trust, Oxford Centre for Diabetes, Endocrinolog; NIHR Biomedical Research Centre, Oxford<br>Jebb, Susan; Univerof Oxford, Primary Care Health Sciences; NIHR Biomedical Research Centre, Oxford
Keywords:	DIABETES & ENDOCRINOLOGY, Obesity, NUTRITION & DIETETICS, Weight Loss, PRIMARY CARE

SCHOLARONE™
Manuscripts

Dietary approaches to the management of type 2 diabetes (DIAMOND): protocol for a randomised feasibility trial [a]

Elizabeth Morris[1,6*]

Paul Aveyard[1,6]

Pamela Dyson[2,6]

Michaela Noreik[1,6]

Clare Bailey[3]

Robin Fox[4]

Kathy Hoffman[5]

Garry D Tan[2,6]

Susan A Jebb[1,6]

*Corresponding Author: Nuffield Department of Primary Care Health Sciences, Radcliffe Primary Care Building, Woodstock Road, Oxford, OX2 6GG, email: elizabeth.morris@phc.ox.ac.uk, tel: 01865 617131

[1] Nuffield Department of Primary Care Health Sciences, University of Oxford, Oxford UK

[2] Oxford Centre for Diabetes, Endocrinology and Metabolism (OCDEM), Churchill Hospital, Oxford University Hospitals NHS Foundation Trust & University of Oxford, Oxford UK

[3] Burnham Health Centre, Slough UK

[4] Bicester Health Centre, Bicester UK

[5] Chiltern CCG, Amersham UK

[6] NIHR Biomedical Research Centre, Oxford UK

[a] This trial is sponsored by the University of Oxford, Clinical Trials and Research Governance, Joint Research Office, Block 60, Churchill Hospital, Old Road, Headington, Oxford, OX3 7LE, UK

Keywords: Diabetes, obesity, diet, weight loss, primary care

Word count: 4694 words

For peer review only

For peer review only - http://bmjopen.bmj.com/site/about/guidelines.xhtml

ABSTRACT

Introduction:

Some clinicians have observed that low-carbohydrate, low-energy diets can improve blood glucose control, with reports of remission from type 2 diabetes in some patients. In clinical trials, support for low-carbohydrate, low-energy diets has been provided by specialist staff and these programmes are unsuitable for widespread deployment in routine primary care. The aim of this trial is to test whether a newly-developed behavioural support programme can effectively deliver a low-energy, low-carbohydrate diet in a primary care setting.

Methods and analysis:

This is a feasibility randomized controlled trial with embedded qualitative study. Thirty adult patients with type 2 diabetes and BMI≥30kg/m^2 in 2-4 general practices will be randomised 2:1 intervention or control and followed up over 12 weeks. The intervention diet comprises eight weeks of a low-carbohydrate food-based diet providing around 800kcal/day, followed by four weeks of weight maintenance. This programme will be delivered by practice nurses, who will also support patients through goal-setting, motivation and self-monitoring across four appointments, and provide a self-help booklet with recipes, shopping lists, and other behavioural support. Primary outcome measures of feasibility will be met if confidence intervals do not cross the following proportions: that 60% of intervention group participants attempt the dietary intervention, healthcare professionals conduct the intervention delivery session with at least 60% of essential elements present, and 60% of participants attend the final follow-up session. Secondary outcome measures will assess process and qualitative measures, as well as exploratory outcomes including change in HbA1c and change in weight.

Ethics and dissemination: This study has been granted ethical approval by the National Research Ethics Service, South Central Oxford B Research Ethics Committee (ref: 18/SC/0071). The study results will inform whether to progress to a full-scale RCT to test the efficacy of offering this programme for patients with type 2 diabetes in primary care.

TRIAL REGISTRATION NUMBER: ISRCTN registry (ISRCTN 62452621)

For peer review only - http://bmjopen.bmj.com/site/about/guidelines.xhtml

ARTICLE SUMMARY

Strengths and limitations of this study

- This study directly addresses a key question regarding the dietary management of patients with type 2 diabetes in routine practice.
- The intervention has been developed by a multi-disciplinary team of academic and clinical professionals with patient involvement to create a rigorous yet practical approach suitable for delivery in routine care
- Quantitative and qualitative analyses will assess the feasibility of the intervention and inform the development of any future full-scale trial
- This study will not demonstrate the clinical effectiveness of this approach and a subsequent definitive trial will be required to consider whether this intervention can lead to weight loss, improved glycaemic control and diabetes remission

INTRODUCTION:

There are an estimated four million people living with diabetes in the UK, with numbers expected to rise to five million by 2025[1]. Globally, almost 15% of all deaths in the 20-79 years age group, are attributable to diabetes[1]. Currently, 10% of the NHS annual budget is spent on diabetes (around £10 billion). When both direct care and indirect costs are considered, this sum rises to £24 billion, and is predicted to rise further to almost £40 billion by 2035[1].

Obesity is one of the strongest risk factors for type 2 diabetes[1], with an estimated 9% increased risk for every 1kg of weight gained[2], while even modest weight loss of 5% can improve glycaemic control[3]. For people with established type 2 diabetes, dietary management remains a cornerstone of high quality care[4], aiming to lower blood glucose levels, and achieve weight loss in those who are overweight[3]. There is strong evidence for the benefit of behavioural interventions in the management of type 2 diabetes; a large, high quality RCT of over 5,000 adults with type 2 diabetes showed that a 12 month intensive behavioural intervention (individual and group meetings to support decreased energy intake and increased physical activity) led to a clinically significant weight loss (mean 8.5 % of initial weight, compared to 0.6% in the control group), and a significant reduction in mean HbA1c (-0.6% (0.55mmol/mol), compared to -0.1% (0.1mmol/mol) (control group)) at 1 year[5]. The benefits of the intervention were still evident at four year follow up (weight loss of 4.7%, and HbA1c reduction of 0.2%(0.2mmol/mol))[6]. Longitudinal data from the UK Prospective Diabetes Study demonstrates that every 1% (11mmol/mol) reduction in HbA1c is

For peer review only - http://bmjopen.bmj.com/site/about/guidelines.xhtml

associated with a 21% reduction in risk of diabetes-related mortality, and 37% reduction in risk of microvascular complications.[7].

Weight loss is associated with reduction in HbA1c. Observational analysis of data from RCTs shows that the odds ratios for a 5-10% and ≥10% weight loss for a clinically significant 0.5% (0.5mmol/mol) reduction in HbA1c were 3.5 (95% CI 2.8 to 4.4) and 5.4 (4.2 to 7.1) respectively[8]. The recent DiRECT trial demonstrated that with a low energy, total diet replacement programme (850 kcal/day formula diet for 3-5 months), delivered within a primary care setting and designed to achieve 15 kg weight loss, 46% of participants in the intervention group (compared to 4% in the control group, OR 19.7 (7.8 to 49.8)) achieved diabetes remission at 12 months[9]. However, total diet replacement with a formula product is not acceptable to some patients, some of whom may prefer a food-based diet. In addition, food-based dietary strategies may help establish a sustainable diet for the longer-term.

There is limited high quality evidence for the optimal macronutrient intake for patients with diabetes wishing to lose weight, or improve their glycaemic control[3 10-12]. Reductions in glycaemic load, through restriction of total carbohydrate and/or changes in the type of carbohydrate to lower the glycaemic index, may have a beneficial effect, due to the direct effect of carbohydrate consumption on post-prandial blood glucose levels[13], leading to a growing interest in carbohydrate-restricted diets for patients with diabetes. This is reflected in the wealth of recent reviews comparing the effects of low- versus high-carbohydrate diets in patients with diabetes[11 14-21]. Intervention studies have reported that low-carbohydrate diets can reduce HbA1c by mean 0.5% (0.5mmol/mol) , and body weight by mean 4.8kg, in people with type 2 diabetes[14 22]. Recent data from systematic reviews demonstrates that these low-carbohydrate diets may be safe and effective in the short-term, yielding greater reductions in HbA1c at 3 (-0.5%, 95% CI -0.7 to -0.2) and 6 months (-0.4%, 95% CI -0.6 to -0.1) than higher-carbohydrate diets[14]. There is no evidence of significant benefit of these diets on HbA1c or weight change at 12 or 24months[14], although the evidence is not precise enough to exclude worthwhile effects on these variables. This apparent diminution in the benefits may be due in part to a decline in dietary adherence over time; additionally, heterogeneity in reporting of medication changes and adjustment for reduction of these in meta-analyses has been suggested to contribute to an under-estimation of effect on glycaemic control[17]. The available evidence has key limitations, including in the variation in definition of what constitutes a “low-carbohydrate” diet, and there remains a lack of pragmatic trials to demonstrate the feasibility of supporting such diets in routine practice. In most trials to date the programme has been delivered by specialist staff, offering in-depth advice and support, which is unrealistic if this is to be delivered at scale.

For peer review only

For peer review only - http://bmjopen.bmj.com/site/about/guidelines.xhtml

In spite of the uncertainties in the evidence base, there is considerable interest from patients and the media in using low-carbohydrate diets in the management of type 2 diabetes[23 24] and some practitioners have begun to recommend patients with type 2 diabetes to follow a low-carbohydrate diet, with or without specific energy restriction[25]. In 2017 the James Lind Alliance identified the role of carbohydrates, dietary change, and how best to support people to achieve these changes, as 3 of the top 10 research priorities in type 2 diabetes[26].

To address this evidence gap, we will conduct a randomised controlled trial to investigate the feasibility of delivering a low-carbohydrate, low-energy, food-based dietary intervention in primary care, to patients with established type 2 diabetes and BMI ≥30kg/m2.

The hypothesis is that compared to usual care, an intervention involving targeted health professional advice, goal setting, and structured materials, will help patients understand and adhere to a low-carbohydrate, low-energy diet that will lead to significant reductions in weight and associated improvements in glycaemic control (or reduction in diabetic medication required to maintain glycaemic control), providing positive reinforcement of the dietary modification. Few people can sustain a low energy diet beyond 8-12 weeks but the low-carbohydrate component offers the potential for sustained glycaemic control. Together with the possibility of returning to a low-energy regimen in the case of weight regain, this dietary strategy could provide the foundation for a sustainable approach to the management of this chronic condition.

The specific aims of the DIAMOND study are to investigate the feasibility of delivering this behavioural and dietary intervention to a population of patients with diabetes in primary care, and determine whether progression to a full scale randomised controlled trial is indicated; to assess achievements against a number of process measures to inform future trial design; and to investigate the potential physical, biochemical, and economic impact of this behavioural and dietary intervention.

METHODS AND ANALYSIS

Design and setting

This feasibility study will be an individually randomised controlled trial, performed in adult patients with type 2 diabetes and a BMI ≥30kg/m^2, recruited from general practices in England. Each participant will be enrolled for 3 months from randomisation to final follow up, and will attend up to

For peer review only - http://bmjopen.bmj.com/site/about/guidelines.xhtml

7 visits. Due to the nature of the intervention, it will not be possible to blind the participants, clinicians delivering the intervention, or some of the study team to the treatment allocation.

Recruitment

Participants will be recruited from between 2 and 4 GP practices for this feasibility study, identified through the regional clinical research network (CRN) and local expressions of interest. The practices will be asked to search their computerised records to identify people who meet the inclusion criteria. Prior to invitation letters being sent, a GP will screen the list of potential participants to ensure that all those identified are medically appropriate to invite to participate in the trial. The practice will then send potential participants an invitation letter asking them to contact the research team if they are interested, and a participant information sheet with further details about the study.

Participants may also be identified opportunistically during routine consultations or resulting from tests performed as part of the NHS Health Checks programme or diabetes annual review appointments. GPs will provide individuals with the invitation letter and information sheet and invite the individual to ring the study team if they are interested in participating.

Interested individuals will respond to the invitation letter and contact the research team by text, email or telephone. During initial telephone contact, a researcher will discuss the study with the individual, and assess self-reported eligibility to participate according to the full inclusion and exclusion criteria as detailed below. They will also establish verbal informed consent for the study team to access the patient's Oxfordshire diabetic eye screening record, to ensure they meet the retinopathy screening inclusion criteria (as this may not always be coded on the GP's electronic notes). Eligible individuals who wish to participate will be booked in for a face-to-face baseline appointment.

Inclusion Criteria

- Participant is willing and able to give informed consent for participation in the study.
- Male or female, aged 18 years or above.
- BMI of $\geq 30 kg/m^2$
- Diagnosed with type 2 diabetes, (as defined by an HbA1c ≥48mmol/mol (6.5%) at time of diagnosis)
- Patients must have undergone diabetic retinopathy screening within the last 12 months

For peer review only - http://bmjopen.bmj.com/site/about/guidelines.xhtml

Exclusion criteria

- History of, or features indicative of, an eating disorder
- Pregnant, breastfeeding, currently undergoing fertility treatment, or planning to become pregnant during the course of the study
- Recent MI or CVA (<3 months)
- Uncontrolled ischaemic heart disease, critical ischaemia, uncontrolled hypertension, uncontrolled cardiac arrhythmia, cardiac conduction abnormality (eg long QT syndrome)
- Cardiac failure (Grade II, III or IV New York Heart Association)
- Renal failure (CKD Stage 4 or 5)
- Active treatment for cancer (other than skin cancer treated with curative intent by local treatment only)
- Intercurrent serious infection at time of recruitment
- Diagnosed with a significant psychiatric disorder or substance abuse
- Serious neurological disorder, including epilepsy
- Recently undergone significant surgery (<6months)
- History of bariatric surgery, including gastric banding
- Are currently using a "fasting"/low-energy diet
- Unwilling to consider any dietary changes
- Unable to understand English
- Are currently using insulin or SGLT2 inhibitor therapy
- Non-proliferative retinopathy level R2 or more severe (ie, any level more severe than "background" non-proliferative diabetic retinopathy, R1), proliferative diabetic retinopathy, or maculopathy.
- HbA1c ≥ 93mmol/mol (10.5%)
- Recruiting physician feels they are inappropriate for recruitment due to any other reason.

Participant flow

Baseline appointments will be conducted with either the practice nurse or a member of the research team at participants' own GP practice, during which informed consent will be sought, eligibility formally assessed, and baseline measurements collected. The participants will then be randomised to one of the two trial arms. Participants allocated to the control arm will continue to an appointment with the practice nurse for "usual care" dietary advice. Participants randomised to receive the intervention will proceed to an appointment with healthcare professionals (practice

For peer review only - http://bmjopen.bmj.com/site/about/guidelines.xhtml

nurse and then GP) at their practice, during which the intervention materials and advice will be delivered, and medication regimes reviewed for those participants on antihypertensive, hypoglycaemic or lipid modifying medications. Those in the intervention arm will subsequently be invited to attend for week 2, 4 and 8 study visits, at which further samples and measurements will be obtained. All participants will be invited for a final 12 week follow up visit, at which time all outcome measures will be repeated. After completion of the 12 week follow-up period, participants will be invited to participate in qualitative focus groups to further explore their experience of this intervention. Participant flow through the study is outlined in Figure 1.

Sample size

The total number of participants recruited for this study will be 30. As this is a feasibility study, it has not been powered to detect a statistically significant difference in efficacy between the arms. In determining thresholds for progression criteria, based on previous trials of a similar nature, we expect at least 75% achievement[27 28]. If so, the 95% confidence interval will exclude 60% and therefore we have set this as the progression criteria for each criterion, requiring a sample size of 30 participants allocated in a 2:1 ratio, intervention:control.

Randomisation

All eligible, consenting participants will be randomised to one of the two trial arms (intervention or control), using permuted block design randomisation with blocks of 3 and 6. Allocation will be stratified by practice. An independent researcher will generate the set of sequences and assign participants to the intervention groups using sequentially numbered sealed envelopes to ensure allocation concealment until interventions are assigned. Due to the nature of this study, it will not be possible to further blind participants, clinicians or some of the study team to the treatment allocation beyond this point.

Intervention

The Diamond programme

The active intervention is a behaviourally informed, low-carbohydrate, low-energy diet delivered by healthcare professionals at a GP practice. It draws upon the motivational value of the relationship between the healthcare professional and the patient, but provides almost all the technical knowledge-transfer through the use of structured materials such as meal plans, thus addressing the uncertainties and lack of confidence expressed by health professionals about giving dietary advice[29].

For peer review only - http://bmjopen.bmj.com/site/about/guidelines.xhtml

The intervention aims to improve patients' adherence to the programme by providing a structured simple behavioural support programme, including goal-setting, planning, feedback and problem solving. It is designed to induce rapid changes in dietary composition and weight loss, followed by a sustainable maintenance programme. The intervention is envisaged to take 10-15 minutes of support from the GP and 80 minutes from the nurse to deliver over four sessions of support (baseline, week 2, week 4, and week 8).

This intervention was designed following the principles of the Person-Based Approach[30]. It involved examining recent systematic reviews, reviewing qualitative studies and process assessments of these types of interventions, and developing the behavioural and dietary intervention programme with a multi-disciplinary team comprising a diabetes-specialist dietician and clinician, primary care physicians, and patient involvement. Two groups of patients with type 2 diabetes, including those who had recently tried variations of a low-carbohydrate, low-energy diet, also met to discuss the intervention and to inform and advise on the dietary and behavioural support components.

The dietary component of this intervention consists of a low-carbohydrate and energy restricted diet (800-1000kcal/day) for 8 weeks, transitioning to a 4 week maintenance period. The core principles include: advice to exclude sugary and starchy foods high in carbohydrates (eg biscuits, confectionery, bread, pasta, potatoes) entirely from their diet (with the exception of dairy and limited fruit intake), strict portion control, and avoiding energy-dense foods. Standardised "healthy eating" advice regarding fresh vegetables, and lean meat and fish, are also included. After the initial 8 week intensive weight loss phase, participants will be advised to gradually increase their energy intake, re-introducing one 'normal-sized' meal at a time, until they remain weight stable, but with guidance on maintaining a sustainable lower-carbohydrate diet in the longer term.

The design of this intervention has been additionally informed by behavioural analysis, identifying domains of the Behaviour Change Wheel[31] and Theoretical Domains Framework[32] to promote successful behaviour change. Utilising these frameworks, the key influencing factors which have been targeted are: psychological capability (including knowledge, skills, decision processes and behavioural regulation), social and physical opportunity (social influences and environmental context), reflective motivation (beliefs about capabilities and consequences, optimism, intentions, goals and reinforcement).

To address these components, the intervention will include:

- A brief behavioural intervention consisting of both dietary and motivational advice, including the rationale behind the intervention, to be delivered by a healthcare

For peer review only

For peer review only - http://bmjopen.bmj.com/site/about/guidelines.xhtml

professional in primary care. The dietary advice includes the essential features of the diet – low energy and low carbohydrate content, as described above.

- Written resources, including advice for meal planning and food selection, and suggested recipes for "real food" choices – to empower patients with the knowledge and skills for decision-making about food.
- Personalised realistic goal setting (e.g. change in weight, HbA1c, number of diabetic medications)
- Diary to record self-assessment of programme adherence.
- Structured healthcare professional follow up, to provide support and contingency planning, and feedback on progress. Additionally, patients will be able to self-monitor their health with the use of home fingerprick-testing blood glucose metres, and BP machines, for additional motivation and monitoring of progress.
- Feedback from healthcare professional on personal changes in health parameters as a consequence of the intervention (changes in weight, BP, HbA1c).

Comparator

Participants randomised to the control arm will receive usual care at their week 0 study visit, comprising a single face-to-face appointment with a healthcare professional, during which they will receive standard dietary and lifestyle information based on the Diabetes UK "what is a healthy balanced diet for diabetes" leaflet. If their managing GP or nurse feels they warrant referral for further diabetic care or input (for example, diabetes education course), or intensification of medications, and would ordinarily have pursued this as part of their routine care outside of the trial, this will be permitted as forming part of "usual care", but will be documented.

Outcomes

Primary

The primary objective of this study is to test the feasibility of a low-carbohydrate, low-energy, behavioural intervention, comprising targeted advice from a health professional combined with written dietary information, to promote weight loss and improved glycaemic control in patients with type 2 diabetes. This feasibility study will then determine whether to progress to a full randomised control trial.

The following progression criteria will determine whether to progress to a full trial:

For peer review only - http://bmjopen.bmj.com/site/about/guidelines.xhtml

1. That 60% of allocated “intervention” group participants attempt the dietary intervention after randomisation.
   (Evaluated by documentation after intervention visit; assessed as the proportion of patients who agree to start the dietary intervention, as recorded by the nurse delivering the intervention session).
2. Fidelity of intervention delivery: That healthcare professionals conduct the intervention delivery session with at least 60% of essential elements present.
   (Evaluated by assessment of audio recorded consultations against a checklist of pre-specified essential elements).
3. That 60% of participants attend the final follow-up session
   (Documentation of final study visit)

Secondary

A mixture of quantitative and qualitative methods will be used to assess process measures and effectiveness measures. This feasibility study is not powered to detect statistical differences in efficacy outcomes but will examine the following parameters in order to test trial procedures, process, resources and management, to aid sample size estimates for a future trial, to determine the most appropriate primary outcome measures for a future trial, and to inform further development of the intervention strategy.

Process Measures*:*

- Percentage of eligible patients, as a proportion of the total population of patiets in a practice, with type 2 diabetes.
- Percentage of people who fulfil the recruitment criteria who accept the invitation to participate
- Proportion of patients who enrol in the study who are deemed to have with “suboptimal control” (HbA1c above the NICE target of ≥7%)
- Participant adherence to the protocol: including, change in dietary composition (low-carbohydrate, energy restricted - assessed using 24 hour dietary recall questionnaires); participants’ self-reported concordance with the intervention; availability of data for outcome measures; attendance at follow up sessions; contamination of the control group (i.e., those who choose to follow the principles of the intervention (i.e. follow a low-carbohydrate diet), despite being allocated to the control group). This will be assessed using 24 hour dietary recall questionnaires to establish change in dietary composition (frequency of carbohydrate consumption, energy restriction) of control group participants.

For peer review only - http://bmjopen.bmj.com/site/about/guidelines.xhtml

For peer review only

- Difference between "baseline" HbA1c value and that used from the latest record as inclusion criteria.
- Serious Adverse Events (SAEs) reported up to the end of the 12 week study participation period.

Exploratory outcomes*:*

- Change in HbA1c - Number of patients previously in diabetic HbA1c range, now in "at risk of diabetes" (6-6.4%) or "diabetes in remission" (<6.0%, off medications) HbA1c range
- Change in fasting glucose, fasting insulin, HOMA-%B, HOMA-%S
- Change in weight
- Change in diabetic medication (number of diabetic medications currently prescribed to the patient; dose of diabetic medications; initiation of new medication during study period; initiation of insulin; initiation of injectable diabetic medication; number of medications stopped or changed during the study period)
- Change in lipid profile – total cholesterol, HDL, triglycerides, calculated non-HDL cholesterol, and total cholesterol:HDL ratio,
- Change in liver function tests (Bilirubin, ALT, AST, ALP, Albumin, AST:ALT)
- Change in blood pressure (systolic, diastolic)
- Change in antihypertensive medication (number of medications started and stopped during study period; dose of antihypertensive medications; initiation of new medication during study period)
- Change in medication prescribing costs (total and diabetic) across study group and total practice diabetic population
- Effect on patient's problem areas in diabetes (PAID) score

Qualitative Measures:

- Experience of and acceptability of the intervention, for patients and healthcare practitioners (assessed using qualitative focus groups following completion of the initial study period).

Measurements

Figure 2 provides a summary of the measurements collected.

Sociodemographic characteristics

Participants will be asked to self-report age, sex and ethnicity, education history and employment status.

For peer review only - http://bmjopen.bmj.com/site/about/guidelines.xhtml

For peer review only

Medical and medication history

Relevant medical history and all current medication will be recorded and checked against the participant's medical record.

Physical measurements

Height will be measured using stadiometers, to the nearest 1cm. Weight will be measured to the nearest 0.1kg using a digital scale (SC-240 MA, Tanita Japan). BMI will be calculated using the standard formula BMI=weight(kg)/height(m)^2. Blood pressure will be measured in triplicate after 5 minutes seated rest, with at least 1 minute between each measurement. All assessors will be trained in standardised methods of taking physical measurements according to the study manual of procedures.

Fasting blood sample

A fasting venous blood sample for HbA1c, fasting insulin, fasting glucose, liver function tests (Bilirubin, ALT, AST, ALP, Albumin) and lipid profile (total cholesterol, triglycerides, HDL, calculated LDL, and total:HDL cholesterol ratio) will be collected at baseline, 8 weeks and 12 weeks. All blood samples will be taken, handled, analysed and disposed of according to standard NHS procedures and local practice policy.

Questionnaires

During study follow-up visits, patients will be asked to complete questionnaires to assess the following measures:

- *Quality of life:* the PAID score (Problem Areas in Diabetes), a 20 item questionnaire measuring problems related to emotions, treatment, food and social support[33], will be measured at baseline and 12 weeks.
- *Dietary composition:* a 24 hour dietary recall questionnaire will be used to assess intake of different food groups at baseline, 2, 8 and 12 weeks.
- *Motivation:* Self-reported motivation and perceptions across domains of diet, health and diabetes control will be assessed using a 6 point questionnaire at baseline and 12 weeks.
- *Self-reported adherence to intervention:* Self-reported adherence to the 3 core components of the dietary intervention will be assessed at 2, 4, 8 and 12 weeks.

For peer review only - http://bmjopen.bmj.com/site/about/guidelines.xhtml

Retention and withdrawal

Each participant will have the right to withdraw from the study at any time. In addition, the investigator may discontinue a participant from the study at any time if they consider it necessary for any reason, including ineligibility (either arising during the study or having been overlooked at screening). If a participant requests to withdraw from the study, it will be explained to them that we would like to use their data collected up to the point at which they have withdrawn from the study, unless they request that we do not do so. The reason for withdrawal will be recorded in the CRF. Withdrawn participants will not be replaced in this feasibility study. To reflect the burden of participation and promote participant retention and complete follow up, participants will be offered a £20 gift card upon attending the 12 week follow-up appointment.

Adverse events

We will record and report all serious adverse events (SAEs) following Good Clinical Practice and standard HRA processes. The duration of the SAE recording period for each participant lasts from their enrolment on to the study, to their completion of the study. We elected not to record other AEs because this process is burdensome for clinicians and participants and we considered that this programme was unlikely to induce significant AEs.

Data management

Data will be recorded on hard copy CRFs and subsequently entered into a web-based data capture system (RedCap, © 2018 Vanderbilt University) with data stored on a secure server hosted by the Primary Care Clinical Trials Unit at the University of Oxford. The system has an inbuilt audit trail facility and ability to run internal validation checks.

Statistical analysis

Primary outcome measures for this study are progression criteria, to inform any future RCT powered to detect an intervention effect. Analysis for progression criteria will utilise data from all participants recruited to the trial. Descriptive statistical methods along with inferential statistics presenting confidence intervals will be used to analyse and report progression criteria.

Secondary outcome measures for this study include process measures, and exploratory effectiveness measures. Both measures will use all collected data, including that from participants who have not

For peer review only - http://bmjopen.bmj.com/site/about/guidelines.xhtml

completed the trial, and from participating practices (where pertaining to proportions of patients identified as eligible, and responding to invitations to participate). Descriptive comparative summary statics (e.g. difference in means or proportions, together with 95% confidence intervals, comparison to baseline and overall change compared to control group) will be used.

Acceptability and experience of the intervention by participants and healthcare professionals will be collected as qualitative data from interviews and focus groups conducted after intervention completion. These data will be qualitatively analysed using thematic analysis, and reported descriptively.

Patient involvement

This study evolved through patient demand in the local area, to know more about dietary options and in particular low-carbohydrate diets for people with type 2 diabetes. We convened two panels of patients with type 2 diabetes, one consisting of members who had recently tried variations of a low-carbohydrate, low-energy diet, to inform and advise on the dietary and behavioural support components of the intervention, the patient materials, and the perceived benefits or burdens of the study for patients. One patient member has subsequently joined the trial management group and will be involved in trial steering decisions during the monitoring and running of the trial, and regarding progressing to a full-scale trial.

Qualitative substudy

The purpose of the nested qualitative sub-study is to explore patients' and healthcare professionals' experience of receiving, enacting and delivering this intervention, and of participating in the study. We will seek the views of up to 10 patient participants from the intervention group, from those who consent to be contacted for the nested qualitative study, and 6-8 healthcare professionals. The patient participants chosen will aim to reflect a range of success with weight loss and glycaemic control, with thought given to not mixing those who have found the programme easy or successful, with those who have not achieved their goals. Additionally, we will aim to capture a breadth of participant characteristics (age, gender, social class, ethnicity, or, for the healthcare professional interviews, the participant's profession). All healthcare professionals who have been involved in the study will be invited to take part. Should more participants volunteer than are required, purposive sampling will be used to ensure both GPs and nurses are represented in the cohort, and to include those who have conducted a greater number of sessions.

For peer review only - http://bmjopen.bmj.com/site/about/guidelines.xhtml

Focus groups will be conducted after all participants have completed their 3 month study follow up period. Written informed consent will be taken before the focus group commences. All focus groups will be audio recorded, and these audio recordings will be transcribed. Patients will be reimbursed for their time and practices will be reimbursed for the healthcare professionals taking time out from their clinical duties to participate.

Focus groups will follow broad topic areas based upon the study objectives, but will encourage participants to discuss their perceptions and experiences freely and in depth. Topics will include: the acceptability of the intervention; views on which components of their treatment group they felt were effective and which were not effective; thoughts about impact on experience of diabetes and its management; thoughts about weight management going forwards. Data will be analysed using thematic analysis.

ETHICS AND DISSEMINATION

The study protocol (V2.0 1.3.18) was reviewed and approved by the South Central Oxford B REC Committee (Ref: 18/SC/0071) and registered on the ISRCTN (62452621). Any protocol modifications will be submitted for review by the research ethics committee and amended at the trial registry.

If the trial proves feasible, according to the specified progression criteria it is planned that the results will inform design of a full-scale randomised trial to test the efficacy of the intervention to improve glycaemic control.

The findings of this feasibility study will be submitted for publication in a peer-reviewed journal, and presented at conferences, to disseminate the results to academic and health professional audiences, and made available to participants and to a wider public on our website at the time of publication.

AUTHOR CONTRIBUTIONS & ACKNOWLEDGEMENTS:

EM, PA and SJ developed the concept for the study and wrote the first draft of the protocol. EM, PA, SJ and PD prepared the study documents and coordinated the HRA and ethics application. EM drafted the manuscript for publication, with input from PA and SJ. All authors were involved in the detailed design of the study, approved the final protocol and manuscript. The authors would like to thank the members of the DIAMOND patient participation panels for their invaluable input and advice throughout all stages of study design.

For peer review only

For peer review only - http://bmjopen.bmj.com/site/about/guidelines.xhtml

FUNDING STATEMENT: PA and SAJ are NIHR Senior Investigators and funded by NIHR CLAHRC Oxford**.** PA, SAJ, GDT, PD, and MN are supported by the NIHR Oxford BRC. This work was supported by the National Institute for Health Research (NIHR) Oxford Biomedical Research Centre (BRC) and the School for Primary Care Research (SPCR) (grant reference number 404).

DISCLAIMER: This report is independent research by the National Institute for Health Research. The views expressed in this publication are those of the author(s) and not necessarily those of the NHS, the University of Oxford, the NIHR or the Department of Health.

COMPETING INTERESTS: PA and SAJ have received research grant funding but no personal renumeration from commercial weight loss companies, but none of these companies have interests in the programme described here. CB is the author of "The 8-week blood sugar diet recipe book", published in 2016. PAD is a member of the PHE/SACNE/DUK committee reviewing evidence on the effects of low carbohydrate diets in people with type 2 diabetes.

DATA STATEMENT: For access to the study data set, a formal request should be sent to the DIAMOND study group. The request will only be considered when the principal results of the study have been published.

CHECKLIST FOR APPROPRIATE REPORTING STATEMENT: SPIRIT 2013 Checklist

REFERENCES:

1. DiabetesUK. Facts and Stats. *Diabetes UK website, wwwdiabetesorguk* 2015
2. Mokdad AH, Ford ES, Bowman BA, et al. Diabetes trends in the U.S.: 1990-1998. *Diabetes care* 2000;23(9):1278-83. doi: 10.2337/diacare.23.9.1278
3. Nield L, Moore H, Hooper L, et al. Dietary advice for treatment of type 2 diabetes mellitus in adults. *Cochrane Database of Systematic Reviews* 2007(3) doi: 10.1002/14651858.CD004097.pub4
4. NICE_Guidance. NG28 Type 2 diabetes in adults: Management. 2015
5. Look_Ahead_Research_Group. Reduction in Weight and Cardiovascular Disease Risk Factors in Individuals With Type 2 Diabetes: One-Year Results of the Look AHEAD Trial. *Diabetes care* 2007;30(6):1374-83. doi: 10.2337/dc07-0048
6. Wing RR. Long-term effects of a lifestyle intervention on weight and cardiovascular risk factors in individuals with type 2 diabetes mellitus: four-year results of the Look AHEAD trial. *Archives of internal medicine* 2010;170(17):1566-75. doi: 10.1001/archinternmed.2010.334 [published Online First: 2010/09/30]
7. Stratton IM, Adler AI, Neil HAW, et al. Association of glycaemia with macrovascular and microvascular complications of type 2 diabetes (UKPDS 35): prospective observational study. *BMJ* 2000;321(7258):405-12. doi: 10.1136/bmj.321.7258.405
8. Wing RR, Lang W, Wadden TA, et al. Benefits of modest weight loss in improving cardiovascular risk factors in overweight and obese individuals with type 2 diabetes. *Diabetes care* 2011;34(7):1481-6. doi: 10.2337/dc10-2415 [published Online First: 2011/05/20]

For peer review only - http://bmjopen.bmj.com/site/about/guidelines.xhtml

9. Lean MEJ, Leslie WS, Barnes AC, et al. Primary care-led weight management for remission of type 2 diabetes (DiRECT): an open-label, cluster-randomised trial. *The Lancet* 2018;391(10120):541-51. doi: 10.1016/S0140-6736(17)33102-1

10. Evert AB, Boucher JL, Cypress M, et al. Nutrition Therapy Recommendations for the Management of Adults With Diabetes. *Diabetes care* 2014;37(Supplement 1):S120-S43. doi: 10.2337/dc14-S120

11. Ajala O, English P, Pinkney J. Systematic review and meta-analysis of different dietary approaches to the management of type 2 diabetes. *The American journal of clinical nutrition* 2013;97(3):505-16. doi: 10.3945/ajcn.112.042457 [published Online First: 2013/02/01]

12. DiabetesUK. Evidence-Based Guidelines for the Prevention and Treatment of Diabetes. *wwwdiabetesorguk* 2011

13. Arora SK, McFarlane SI. The case for low carbohydrate diets in diabetes management. *Nutrition & Metabolism* 2005;2:16-16. doi: 10.1186/1743-7075-2-16

14. Sainsbury E, Kizirian NV, Partridge SR, et al. Effect of dietary carbohydrate restriction on glycemic control in adults with diabetes: A systematic review and meta-analysis. *Diabetes research and clinical practice* 2018;139:239-52. doi: https://doi.org/10.1016/j.diabres.2018.02.026

15. Dyson P. Low Carbohydrate Diets and Type 2 Diabetes: What is the Latest Evidence? *Diabetes Therapy* 2015;6(4):411-24. doi: 10.1007/s13300-015-0136-9

16. van Wyk HJ, Davis RE, Davies JS. A critical review of low-carbohydrate diets in people with Type 2 diabetes. *Diabetic medicine : a journal of the British Diabetic Association* 2016;33(2):148-57. doi: 10.1111/dme.12964 [published Online First: 2015/09/29]

17. Snorgaard O, Poulsen GM, Andersen HK, et al. Systematic review and meta-analysis of dietary carbohydrate restriction in patients with type 2 diabetes. *BMJ Open Diabetes Research & Care* 2017;5(1) doi: 10.1136/bmjdrc-2016-000354

18. Meng Y, Bai H, Wang S, et al. Efficacy of low carbohydrate diet for type 2 diabetes mellitus management: A systematic review and meta-analysis of randomized controlled trials. *Diabetes research and clinical practice* 2017;131:124-31. doi: 10.1016/j.diabres.2017.07.006 [published Online First: 2017/07/28]

19. Huntriss R, Campbell M, Bedwell C. The interpretation and effect of a low-carbohydrate diet in the management of type 2 diabetes: a systematic review and meta-analysis of randomised controlled trials. *European journal of clinical nutrition* 2018;72(3):311-25. doi: 10.1038/s41430-017-0019-4 [published Online First: 2017/12/23]

20. Fan Y DH, Chen G, Mao X, Liu C. Effects of low carbohydrate diets in individuals with type 2 diabetes: systematic review and meta-analysis. *Int J Clin Exp Med* 2016;9(6):11166-74.

21. Castaneda-Gonzalez LM, Bacardi Gascon M, Jimenez Cruz A. Effects of low carbohydrate diets on weight and glycemic control among type 2 diabetes individuals: a systemic review of RCT greater than 12 weeks. *Nutricion hospitalaria* 2011;26(6):1270-6. doi: 10.1590/s0212-16112011000600013 [published Online First: 2012/03/14]

22. Dyson P. Low Carbohydrate Diets and Type 2 Diabetes: What is the Latest Evidence? *Additional data via personal communication. Diabetes Therapy* 2015;6(4):411-24. doi: 10.1007/s13300-015-0136-9

23. Whitington AaI. "Fixing Dad". *Whitington Brothers Films; Broadcast BBC 2 24072016* 2016

24. Rangan Chatterjee RP, Tim Harcourt, Carrie Britton, Jack Rampling. Doctor in the House. *Studio Lambert Production; Broadcast BBC2 6116* 2015

25. Unwin M, Unwin, J. Low carbohydrate diet to achieve weight loss and improve HbA1c in type 2 diabetes and pre-diabetes: experience from one general practice,. *Practical Diabetes* 2014;31(2)

26. JLA. James Lind Alliance Priority Setting partnerships: Diabetes (Type 2) Top 10. *http://wwwjlanihracuk/priority-setting-partnerships/diabetes-type-2/top-10-uncertaintieshtm (accessed July 2018)* 2017

For peer review only - http://bmjopen.bmj.com/site/about/guidelines.xhtml

27. Leslie WS, Ford I, Sattar N, et al. The Diabetes Remission Clinical Trial (DiRECT): protocol for a cluster randomised trial. *BMC family practice* 2016;17(1):1-10. doi: 10.1186/s12875-016-0406-2

28. Aveyard P, Lewis A, Tearne S, et al. Screening and brief intervention for obesity in primary care: a parallel, two-arm, randomised trial. *Lancet* 2016 doi: 10.1016/s0140-6736(16)31893-1 [published Online First: 2017/01/04]

29. Huang J, Yu H, Marin E, et al. Physicians' weight loss counseling in two public hospital primary care clinics. *Academic medicine : journal of the Association of American Medical Colleges* 2004;79(2):156-61. [published Online First: 2004/01/28]

30. Yardley L, Morrison L, Bradbury K, et al. The Person-Based Approach to Intervention Development: Application to Digital Health-Related Behavior Change Interventions. *J Med Internet Res* 2015;17(1):e30. doi: 10.2196/jmir.4055

31. Michie S, van Stralen MM, West R. The behaviour change wheel: a new method for characterising and designing behaviour change interventions. *Implementation science : IS* 2011;6:42. doi: 10.1186/1748-5908-6-42 [published Online First: 2011/04/26]

32. Michie S, Johnston M, Abraham C, et al. Making psychological theory useful for implementing evidence based practice: a consensus approach. *Quality & safety in health care* 2005;14(1):26-33. doi: 10.1136/qshc.2004.011155 [published Online First: 2005/02/05]

33. Polonsky WH, Anderson BJ, Lohrer PA, et al. Assessment of diabetes-related distress. *Diabetes care* 1995;18(6):754-60. [published Online First: 1995/06/01]

For peer review only

For peer review only - http://bmjopen.bmj.com/site/about/guidelines.xhtml

Figure 1: Participant flow through the study

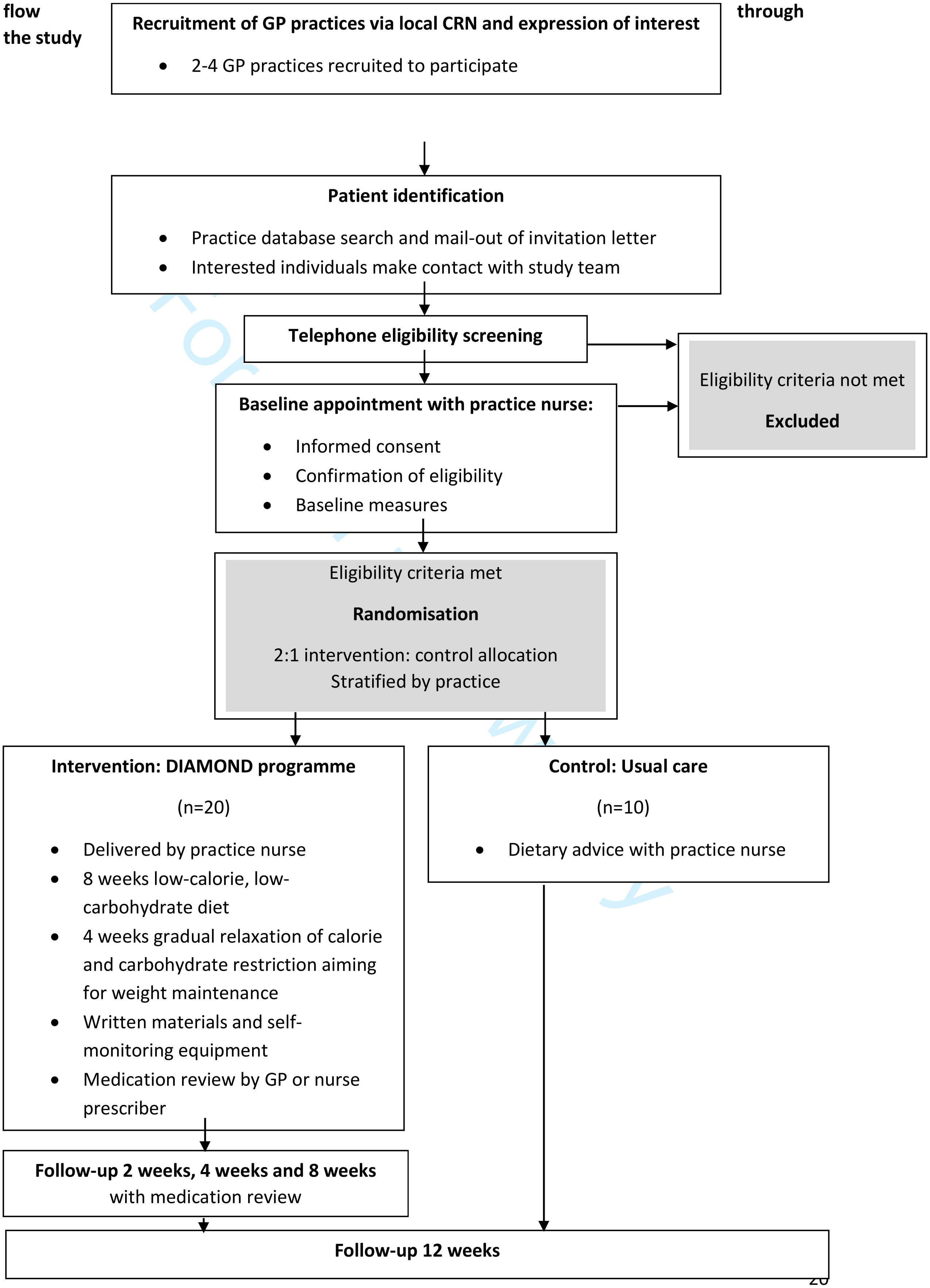

For peer review only - http://bmjopen.bmj.com/site/about/guidelines.xhtml

Figure 2: Schedule of measurements

	STUDY PERIOD					
	Enrolment	Allocation	Study Visits			Follow up
**TIMEPOINT**	***Baseline***	**0**	***Week 2***	***Week 4***	***Week 8***	***Week 12***
**ENROLMENT:**						
Eligibility screen	X					
Informed consent	X					
Randomisation	X					
**INTERVENTION GROUPS:**						
DIAMOND programme		X	X	X	X	X
Usual Care		X	X			X
**ASSESSMENTS:**						
Demographics	X					
Medical history	X					X
Medication review	X		X	X	X	X
Weight	X		X	X	X	X
Height	X		X	X	X	X
Blood pressure	X		X	X	X	X
Fasting blood sample	X				X	X
Adverse event assessment			X	X	X	X
24 hour dietary recall questionnaire	X		X		X	X
Motivation assessment	X					X
Problem Areas in Diabetes (PAID) score	X					X
Self-reported adherence to intervention	X		X	X	X	X

For peer review only

For peer review only - http://bmjopen.bmj.com/site/about/guidelines.xhtml

Figure 1: Participant flow through the study

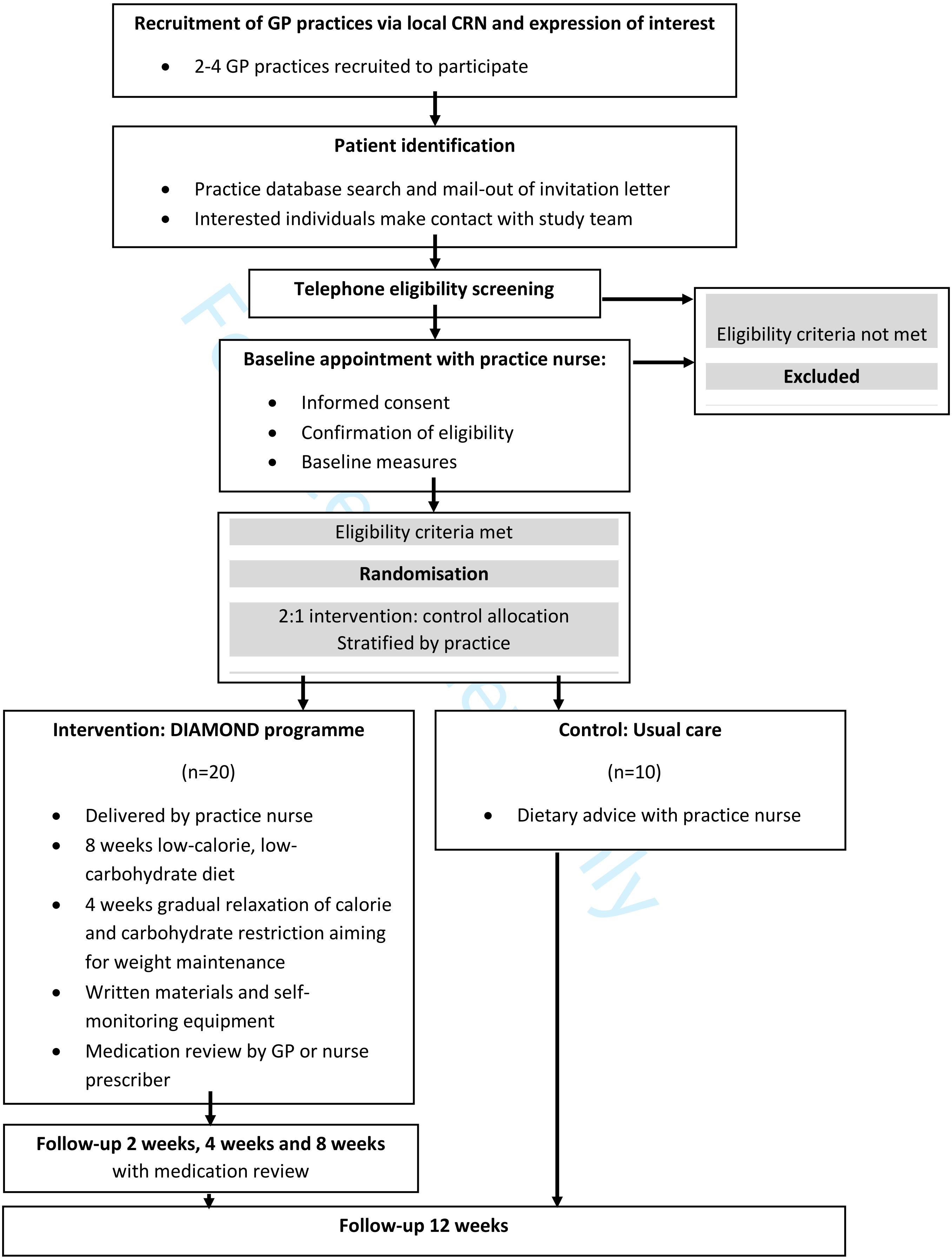

Figure 2: Schedule of measurements

	STUDY PERIOD					
	**Enrolment**	**Allocation**	**Study Visits**			**Follow up**
**TIMEPOINT**	***Baseline***	**0**	***Week 2***	***Week 4***	***Week 8***	***Week 12***
**ENROLMENT:**						
Eligibility screen	X					
Informed consent	X					
Randomisation	X					
**INTERVENTION GROUPS:**						
DIAMOND programme		X	X	X	X	X
Usual Care		X	X			X
**ASSESSMENTS:**						
Demographics	X					
Medical history	X					X
Medication review	X		X	X	X	X
Weight	X		X	X	X	X
Height	X		X	X	X	X
Blood pressure	X		X	X	X	X
Fasting blood sample	X				X	X
Adverse event assessment			X	X	X	X
24 hour dietary recall questionnaire	X		X		X	X
Motivation assessment	X					X
Problem Areas in Diabetes (PAID) score	X					X
Self-reported adherence to intervention	X		X	X	X	X

For peer review only

SPIRIT

STANDARD PROTOCOL ITEMS: RECOMMENDATIONS FOR INTERVENTIONAL TRIALS

SPIRIT 2013 Checklist: Recommended items to address in a clinical trial protocol and related documents*

Section/item	Item No	Description	Addressed on page number
**Administrative information**			
Title	1	Descriptive title identifying the study design, population, interventions, and, if applicable, trial acronym	1____________
Trial registration	2a	Trial identifier and registry name. If not yet registered, name of intended registry	1_____________
	2b	All items from the World Health Organization Trial Registration Data Set	Y_____________
Protocol version	3	Date and version identifier	_16____________
Funding	4	Sources and types of financial, material, and other support	17_____________
Roles and responsibilities	5a	Names, affiliations, and roles of protocol contributors	1,16,17_________
	5b	Name and contact information for the trial sponsor	1
	5c	Role of study sponsor and funders, if any, in study design; collection, management, analysis, and interpretation of data; writing of the report; and the decision to submit the report for publication, including whether they will have ultimate authority over any of these activities	17
	5d	Composition, roles, and responsibilities of the coordinating centre, steering committee, endpoint adjudication committee, data management team, and other individuals or groups overseeing the trial, if applicable (see Item 21a for data monitoring committee)	N/A___________

For peer review only

For peer review only - http://bmjopen.bmj.com/site/about/guidelines.xhtml

Introduction

Background and rationale	6a	Description of research question and justification for undertaking the trial, including summary of relevant studies (published and unpublished) examining benefits and harms for each intervention	3-5____________
	6b	Explanation for choice of comparators	3-5_____________
Objectives	7	Specific objectives or hypotheses	5______________
Trial design	8	Description of trial design including type of trial (eg, parallel group, crossover, factorial, single group), allocation ratio, and framework (eg, superiority, equivalence, noninferiority, exploratory)	5______________

Methods: Participants, interventions, and outcomes

Study setting	9	Description of study settings (eg, community clinic, academic hospital) and list of countries where data will be collected. Reference to where list of study sites can be obtained	5______________
Eligibility criteria	10	Inclusion and exclusion criteria for participants. If applicable, eligibility criteria for study centres and individuals who will perform the interventions (eg, surgeons, psychotherapists)	6-7____________
Interventions	11a	Interventions for each group with sufficient detail to allow replication, including how and when they will be administered	8-9_____________
	11b	Criteria for discontinuing or modifying allocated interventions for a given trial participant (eg, drug dose change in response to harms, participant request, or improving/worsening disease)	14______________
	11c	Strategies to improve adherence to intervention protocols, and any procedures for monitoring adherence (eg, drug tablet return, laboratory tests)	8,14__________
	11d	Relevant concomitant care and interventions that are permitted or prohibited during the trial	9_____________
Outcomes	12	Primary, secondary, and other outcomes, including the specific measurement variable (eg, systolic blood pressure), analysis metric (eg, change from baseline, final value, time to event), method of aggregation (eg, median, proportion), and time point for each outcome. Explanation of the clinical relevance of chosen efficacy and harm outcomes is strongly recommended	10-12 ______________
Participant timeline	13	Time schedule of enrolment, interventions (including any run-ins and washouts), assessments, and visits for participants. A schematic diagram is highly recommended (see Figure)	20______________

For peer review only - http://bmjopen.bmj.com/site/about/guidelines.xhtml

Sample size	14	Estimated number of participants needed to achieve study objectives and how it was determined, including clinical and statistical assumptions supporting any sample size calculations	8_____________
Recruitment	15	Strategies for achieving adequate participant enrolment to reach target sample size	14_____________

Methods: Assignment of interventions (for controlled trials)

Allocation:			
Sequence generation	16a	Method of generating the allocation sequence (eg, computer-generated random numbers), and list of any factors for stratification. To reduce predictability of a random sequence, details of any planned restriction (eg, blocking) should be provided in a separate document that is unavailable to those who enrol participants or assign interventions	8_____________
Allocation concealment mechanism	16b	Mechanism of implementing the allocation sequence (eg, central telephone; sequentially numbered, opaque, sealed envelopes), describing any steps to conceal the sequence until interventions are assigned	_8____________
Implementation	16c	Who will generate the allocation sequence, who will enrol participants, and who will assign participants to interventions	_8____________
Blinding (masking)	17a	Who will be blinded after assignment to interventions (eg, trial participants, care providers, outcome assessors, data analysts), and how	8_____________
	17b	If blinded, circumstances under which unblinding is permissible, and procedure for revealing a participant's allocated intervention during the trial	_8____________

Methods: Data collection, management, and analysis

Data collection methods	18a	Plans for assessment and collection of outcome, baseline, and other trial data, including any related processes to promote data quality (eg, duplicate measurements, training of assessors) and a description of study instruments (eg, questionnaires, laboratory tests) along with their reliability and validity, if known. Reference to where data collection forms can be found, if not in the protocol	10-14_________
	18b	Plans to promote participant retention and complete follow-up, including list of any outcome data to be collected for participants who discontinue or deviate from intervention protocols	_14____________

For peer review only - http://bmjopen.bmj.com/site/about/guidelines.xhtml

Data management	19	Plans for data entry, coding, security, and storage, including any related processes to promote data quality (eg, double data entry; range checks for data values). Reference to where details of data management procedures can be found, if not in the protocol	14_____________
Statistical methods	20a	Statistical methods for analysing primary and secondary outcomes. Reference to where other details of the statistical analysis plan can be found, if not in the protocol	_14-15_________
	20b	Methods for any additional analyses (eg, subgroup and adjusted analyses)	15_____________
	20c	Definition of analysis population relating to protocol non-adherence (eg, as randomised analysis), and any statistical methods to handle missing data (eg, multiple imputation)	_____________
**Methods: Monitoring**			
Data monitoring	21a	Composition of data monitoring committee (DMC); summary of its role and reporting structure; statement of whether it is independent from the sponsor and competing interests; and reference to where further details about its charter can be found, if not in the protocol. Alternatively, an explanation of why a DMC is not needed	n/a___________
	21b	Description of any interim analyses and stopping guidelines, including who will have access to these interim results and make the final decision to terminate the trial	14___________
Harms	22	Plans for collecting, assessing, reporting, and managing solicited and spontaneously reported adverse events and other unintended effects of trial interventions or trial conduct	14_____________
Auditing	23	Frequency and procedures for auditing trial conduct, if any, and whether the process will be independent from investigators and the sponsor	14_____________
**Ethics and dissemination**			
Research ethics approval	24	Plans for seeking research ethics committee/institutional review board (REC/IRB) approval	16_____________
Protocol amendments	25	Plans for communicating important protocol modifications (eg, changes to eligibility criteria, outcomes, analyses) to relevant parties (eg, investigators, REC/IRBs, trial participants, trial registries, journals, regulators)	16_____________

For peer review only - http://bmjopen.bmj.com/site/about/guidelines.xhtml

Consent or assent	26a	Who will obtain informed consent or assent from potential trial participants or authorised surrogates, and how (see Item 32)	_7_____________
	26b	Additional consent provisions for collection and use of participant data and biological specimens in ancillary studies, if applicable	_7_____________
Confidentiality	27	How personal information about potential and enrolled participants will be collected, shared, and maintained in order to protect confidentiality before, during, and after the trial	_7_____________
Declaration of interests	28	Financial and other competing interests for principal investigators for the overall trial and each study site	_17____________
Access to data	29	Statement of who will have access to the final trial dataset, and disclosure of contractual agreements that limit such access for investigators	17_____________
Ancillary and post-trial care	30	Provisions, if any, for ancillary and post-trial care, and for compensation to those who suffer harm from trial participation	_n/a___________
Dissemination policy	31a	Plans for investigators and sponsor to communicate trial results to participants, healthcare professionals, the public, and other relevant groups (eg, via publication, reporting in results databases, or other data sharing arrangements), including any publication restrictions	16_____________
	31b	Authorship eligibility guidelines and any intended use of professional writers	______________
	31c	Plans, if any, for granting public access to the full protocol, participant-level dataset, and statistical code	17_____________
**Appendices**			
Informed consent materials	32	Model consent form and other related documentation given to participants and authorised surrogates	______________
Biological specimens	33	Plans for collection, laboratory evaluation, and storage of biological specimens for genetic or molecular analysis in the current trial and for future use in ancillary studies, if applicable	______________

*It is strongly recommended that this checklist be read in conjunction with the SPIRIT 2013 Explanation & Elaboration for important clarification on the items. Amendments to the protocol should be tracked and dated. The SPIRIT checklist is copyrighted by the SPIRIT Group under the Creative Commons "Attribution-NonCommercial-NoDerivs 3.0 Unported" license.

For peer review only - http://bmjopen.bmj.com/site/about/guidelines.xhtml

BMJ Open

Dietary approaches to the management of type 2 diabetes (DIAMOND): protocol for a randomised feasibility trial

Journal:	*BMJ Open*
Manuscript ID	bmjopen-2018-026460.R1
Article Type:	Protocol
Date Submitted by the Author:	09-Nov-2018
Complete List of Authors:	Morris, Elizabeth; University of Oxford, Nuffield Department of Primary Care Health Sciences; NIHR Biomedical Research Centre, Oxford<br>Aveyard, Paul; University of Oxford, Primary Care Health Sciences; NIHR Biomedical Research Centre, Oxford<br>Dyson, Pamela; University of Oxford , Oxford Centre for Diabetes, Endocrinology and Metabolism; NIHR Biomedical Research Centre, Oxford<br>Noreik, Michaela; University of Oxford Department of Primary Care Health Sciences; NIHR Biomedical Research Centre, Oxford<br>Bailey, Clare; Burnham Health Centre<br>Fox, Robin; Bicester Health Centre<br>Hoffman, Kathy; NHS Chiltern Clinical Commissioning Group<br>Tan, Garry; Oxford University Hospitals NHS Foundation Trust, Oxford Centre for Diabetes, Endocrinolog; NIHR Biomedical Research Centre, Oxford<br>Jebb, Susan; Univerof Oxford, Primary Care Health Sciences; NIHR Biomedical Research Centre, Oxford
**Primary Subject Heading**:	Diabetes and endocrinology
Secondary Subject Heading:	General practice / Family practice, Nutrition and metabolism, Patient-centred medicine
Keywords:	DIABETES & ENDOCRINOLOGY, Obesity, NUTRITION & DIETETICS, Weight Loss, PRIMARY CARE

SCHOLARONE™
Manuscripts

Dietary approaches to the management of type 2 diabetes (DIAMOND): protocol for a randomised feasibility trial [a]

Elizabeth Morris[1,6*]

Paul Aveyard[1,6]

Pamela Dyson[2,6]

Michaela Noreik[1,6]

Clare Bailey[3]

Robin Fox[4]

Kathy Hoffman[5]

Garry D Tan[2,6]

Susan A Jebb[1,6]

*Corresponding Author: Nuffield Department of Primary Care Health Sciences, Radcliffe Primary Care Building, Woodstock Road, Oxford, OX2 6GG, email: elizabeth.morris@phc.ox.ac.uk, tel: 01865 617131

[1] Nuffield Department of Primary Care Health Sciences, University of Oxford, Oxford UK

[2] Oxford Centre for Diabetes, Endocrinology and Metabolism (OCDEM), Churchill Hospital, Oxford University Hospitals NHS Foundation Trust & University of Oxford, Oxford UK

[3] Burnham Health Centre, Slough UK

[4] Bicester Health Centre, Bicester UK

[5] Chiltern CCG, Amersham UK

[6] NIHR Biomedical Research Centre, Oxford UK

[a] This trial is sponsored by the University of Oxford, Clinical Trials and Research Governance, Joint Research Office, Block 60, Churchill Hospital, Old Road, Headington, Oxford, OX3 7LE, UK

Keywords: Diabetes, obesity, diet, weight loss, primary care

Word count: 4694 words

For peer review only

ABSTRACT

Introduction:

Some clinicians have observed that low-carbohydrate, low-energy diets can improve blood glucose control, with reports of remission from type 2 diabetes in some patients. In clinical trials, support for low-carbohydrate, low-energy diets has been provided by specialist staff and these programmes are unsuitable for widespread deployment in routine primary care. The aim of this trial is to test whether a newly-developed behavioural support programme can effectively deliver a low-energy, low-carbohydrate diet in a primary care setting.

Methods and analysis:

This is a feasibility randomized controlled trial with embedded qualitative study. Thirty adult patients with type 2 diabetes and BMI≥30kg/m^2 in 2-4 general practices will be randomised 2:1 intervention or control and followed up over 12 weeks. The intervention diet comprises eight weeks of a low-carbohydrate food-based diet providing around 800kcal/day, followed by four weeks of weight maintenance. This programme will be delivered by practice nurses, who will also support patients through goal-setting, motivation and self-monitoring across four appointments, and provide a self-help booklet with recipes, shopping lists, and other behavioural support. Primary outcome measures of feasibility will be met if confidence intervals do not cross the following proportions: that 60% of intervention group participants attempt the dietary intervention, healthcare professionals conduct the intervention delivery session with at least 60% of essential elements present, and 60% of participants attend the final follow-up session. Secondary outcome measures will assess process and qualitative measures, as well as exploratory outcomes including change in HbA1c and change in weight.

Ethics and dissemination: This study has been granted ethical approval by the National Research Ethics Service, South Central Oxford B Research Ethics Committee (ref: 18/SC/0071). The study results will inform whether to progress to a full-scale RCT to test the efficacy of offering this programme for patients with type 2 diabetes in primary care.

TRIAL REGISTRATION NUMBER: ISRCTN registry (ISRCTN 62452621)

For peer review only

For peer review only - http://bmjopen.bmj.com/site/about/guidelines.xhtml

ARTICLE SUMMARY

Strengths and limitations of this study

- This study directly addresses a key question regarding the dietary management of patients with type 2 diabetes in routine practice.
- The intervention has been developed by a multi-disciplinary team of academic and clinical professionals with patient involvement to create a rigorous yet practical approach suitable for delivery in routine care
- Quantitative and qualitative analyses will assess the feasibility of the intervention and inform the development of any future full-scale trial
- This study will not demonstrate the clinical effectiveness of this approach and a subsequent definitive trial will be required to consider whether this intervention can lead to weight loss, improved glycaemic control and diabetes remission

INTRODUCTION:

There are an estimated four million people living with diabetes in the UK, with numbers expected to rise to five million by 2025[1]. Globally, almost 15% of all deaths in the 20-79 years age group, are attributable to diabetes[1]. Currently, 10% of the NHS annual budget is spent on diabetes (around £10 billion). When both direct care and indirect costs are considered, this sum rises to £24 billion, and is predicted to rise further to almost £40 billion by 2035[1].

Obesity is one of the strongest risk factors for type 2 diabetes[1], with an estimated 9% increased risk for every 1kg of weight gained[2], while even modest weight loss of 5% can improve glycaemic control[3]. For people with established type 2 diabetes, dietary management remains a cornerstone of high quality care[4], aiming to lower blood glucose levels, and achieve weight loss in those who are overweight[3]. There is strong evidence for the benefit of behavioural interventions in the management of type 2 diabetes; a large, high quality RCT of over 5,000 adults with type 2 diabetes showed that a 12 month intensive behavioural intervention (individual and group meetings to support decreased energy intake and increased physical activity) led to a clinically significant weight loss (mean 8.5 % of initial weight, compared to 0.6% in the control group), and a significant reduction in mean HbA1c (-0.6% (0.55mmol/mol), compared to -0.1% (0.1mmol/mol) (control group)) at 1 year[5]. The benefits of the intervention were still evident at four year follow up (weight loss of 4.7%, and HbA1c reduction of 0.2%(0.2mmol/mol))[6]. Longitudinal data from the UK Prospective Diabetes Study demonstrates that every 1% (11mmol/mol) reduction in HbA1c is

For peer review only - http://bmjopen.bmj.com/site/about/guidelines.xhtml

associated with a 21% reduction in risk of diabetes-related mortality, and 37% reduction in risk of microvascular complications.[7].

Weight loss is associated with reduction in HbA1c. Observational analysis of data from RCTs shows that the odds ratios for a 5-10% and ≥10% weight loss for a clinically significant 0.5% (0.5mmol/mol) reduction in HbA1c were 3.5 (95% CI 2.8 to 4.4) and 5.4 (4.2 to 7.1) respectively[8]. The recent DiRECT trial demonstrated that with a low energy, total diet replacement programme (850 kcal/day formula diet for 3-5 months), delivered within a primary care setting and designed to achieve 15 kg weight loss, 46% of participants in the intervention group (compared to 4% in the control group, OR 19.7 (7.8 to 49.8)) achieved diabetes remission at 12 months[9]. However, total diet replacement with a formula product is not acceptable to some patients, some of whom may prefer a food-based diet. In addition, food-based dietary strategies may help establish a sustainable diet for the longer-term.

There is limited high quality evidence for the optimal macronutrient intake for patients with diabetes wishing to lose weight, or improve their glycaemic control[3 10-12]. Reductions in glycaemic load, through restriction of total carbohydrate and/or changes in the type of carbohydrate to lower the glycaemic index, may have a beneficial effect, due to the direct effect of carbohydrate consumption on post-prandial blood glucose levels[13], leading to a growing interest in carbohydrate-restricted diets for patients with diabetes. This is reflected in the wealth of recent reviews comparing the effects of low- versus high-carbohydrate diets in patients with diabetes[11 14-21]. Intervention studies have reported that low-carbohydrate diets can reduce HbA1c by mean 0.5% (0.5mmol/mol) , and body weight by mean 4.8kg, in people with type 2 diabetes[14 22]. Recent data from systematic reviews demonstrates that these low-carbohydrate diets may be safe and effective in the short-term, yielding greater reductions in HbA1c at 3 (-0.5%, 95% CI -0.7 to -0.2) and 6 months (-0.4%, 95% CI -0.6 to -0.1) than higher-carbohydrate diets[14]. There is no evidence of significant benefit of these diets on HbA1c or weight change at 12 or 24months[14], although the evidence is not precise enough to exclude worthwhile effects on these variables. This apparent diminution in the benefits may be due in part to a decline in dietary adherence over time; additionally, heterogeneity in reporting of medication changes and adjustment for reduction of these in meta-analyses has been suggested to contribute to an under-estimation of effect on glycaemic control[17]. The available evidence has key limitations, including in the variation in definition of what constitutes a “low-carbohydrate” diet, and there remains a lack of pragmatic trials to demonstrate the feasibility of supporting such diets in routine practice. In most trials to date the programme has been delivered by specialist staff, offering in-depth advice and support, which is unrealistic if this is to be delivered at scale.

For peer review only

For peer review only - http://bmjopen.bmj.com/site/about/guidelines.xhtml

In spite of the uncertainties in the evidence base, there is considerable interest from patients and the media in using low-carbohydrate diets in the management of type 2 diabetes[23 24] and some practitioners have begun to recommend patients with type 2 diabetes to follow a low-carbohydrate diet, with or without specific energy restriction[25]. In 2017 the James Lind Alliance identified the role of carbohydrates, dietary change, and how best to support people to achieve these changes, as 3 of the top 10 research priorities in type 2 diabetes[26].

To address this evidence gap, we will conduct a randomised controlled trial to investigate the feasibility of delivering a low-carbohydrate, low-energy, food-based dietary intervention in primary care, to patients with established type 2 diabetes and BMI ≥30kg/m2.

The hypothesis is that compared to usual care, an intervention involving targeted health professional advice, goal setting, and structured materials, will help patients understand and adhere to a low-carbohydrate, low-energy diet that will lead to significant reductions in weight and associated improvements in glycaemic control (or reduction in diabetic medication required to maintain glycaemic control), providing positive reinforcement of the dietary modification. Few people can maintain a low energy diet indefinitely (the mean duration of low calorie liquid diet product in the DiRECT study was 16 weeks[9]), but a low-carbohydrate component offers the potential for sustained glycaemic control. Together with the possibility of returning to a low-energy regimen in the case of weight regain, this dietary strategy could provide the foundation for a sustainable approach to the management of this chronic condition.

The specific aims of the DIAMOND study are to investigate the feasibility of delivering this behavioural and dietary intervention to a population of patients with diabetes in primary care, and determine whether progression to a full scale randomised controlled trial is indicated; to assess achievements against a number of process measures to inform future trial design; and to investigate the potential physical, biochemical, and economic impact of this behavioural and dietary intervention.

METHODS AND ANALYSIS

Design and setting

This feasibility study will be an individually randomised controlled trial, performed in adult patients with type 2 diabetes and a BMI ≥30kg/m^2, recruited from general practices in England. Each participant will be enrolled for 3 months from randomisation to final follow up, and will attend up to

For peer review only - http://bmjopen.bmj.com/site/about/guidelines.xhtml

7 visits. Due to the nature of the intervention, it will not be possible to blind the participants, clinicians delivering the intervention, or some of the study team to the treatment allocation.

Recruitment

Participants will be recruited from between 2 and 4 GP practices for this feasibility study, identified through the regional clinical research network (CRN) and local expressions of interest. The practices will be asked to search their computerised records to identify people who meet the inclusion criteria. Prior to invitation letters being sent, a GP will screen the list of potential participants to ensure that all those identified are medically appropriate to invite to participate in the trial. The practice will then send potential participants an invitation letter asking them to contact the research team if they are interested, and a participant information sheet with further details about the study.

Participants may also be identified opportunistically during routine consultations or resulting from tests performed as part of the NHS Health Checks programme or diabetes annual review appointments. GPs will provide individuals with the invitation letter and information sheet and invite the individual to ring the study team if they are interested in participating.

Interested individuals will respond to the invitation letter and contact the research team by text, email or telephone. During initial telephone contact, a researcher will discuss the study with the individual, and assess self-reported eligibility to participate according to the full inclusion and exclusion criteria as detailed below. They will also establish verbal informed consent for the study team to access the patient's Oxfordshire diabetic eye screening record, to ensure they meet the retinopathy screening inclusion criteria (as this may not always be coded on the GP's electronic notes). Eligible individuals who wish to participate will be booked in for a face-to-face baseline appointment.

Inclusion Criteria

- Participant is willing and able to give informed consent for participation in the study.
- Male or female, aged 18 years or above.
- BMI of $\geq 30 kg/m^2$
- Diagnosed with type 2 diabetes, (as defined by an HbA1c ≥48mmol/mol (6.5%) at time of diagnosis)
- Patients must have undergone diabetic retinopathy screening within the last 12 months

For peer review only - http://bmjopen.bmj.com/site/about/guidelines.xhtml

Exclusion criteria

- History of, or features indicative of, an eating disorder
- Pregnant, breastfeeding, currently undergoing fertility treatment, or planning to become pregnant during the course of the study
- Recent MI or CVA (<3 months)
- Uncontrolled ischaemic heart disease, critical ischaemia, uncontrolled hypertension, uncontrolled cardiac arrhythmia, cardiac conduction abnormality (eg long QT syndrome)
- Cardiac failure (Grade II, III or IV New York Heart Association)
- Renal failure (CKD Stage 4 or 5)
- Active treatment for cancer (other than skin cancer treated with curative intent by local treatment only)
- Intercurrent serious infection at time of recruitment
- Diagnosed with a significant psychiatric disorder or substance abuse
- Serious neurological disorder, including epilepsy
- Recently undergone significant surgery (<6months)
- History of bariatric surgery, including gastric banding
- Are currently using a “fasting”/low-energy diet
- Unwilling to consider any dietary changes
- Unable to understand English
- Are currently using insulin or SGLT2 inhibitor therapy
- Non-proliferative retinopathy level R2 or more severe (ie, any level more severe than “background” non-proliferative diabetic retinopathy, R1), proliferative diabetic retinopathy, or maculopathy.
- HbA1c ≥ 93mmol/mol (10.5%)
- Recruiting physician feels they are inappropriate for recruitment due to any other reason.

Participant flow

Baseline appointments will be conducted with either the practice nurse or a member of the research team at participants’ own GP practice, during which informed consent will be sought, eligibility formally assessed, and baseline measurements collected. The participants will then be randomised to one of the two trial arms. Participants allocated to the control arm will continue to an appointment with the practice nurse for “usual care” dietary advice. Participants randomised to receive the intervention will proceed to an appointment with healthcare professionals (practice

For peer review only

For peer review only - http://bmjopen.bmj.com/site/about/guidelines.xhtml

nurse and then GP) at their practice, during which the intervention materials and advice will be delivered, and medication regimes reviewed for those participants on antihypertensive, hypoglycaemic or lipid modifying medications. Those in the intervention arm will subsequently be invited to attend for week 2, 4 and 8 study visits, at which further samples and measurements will be obtained. All participants will be invited for a final 12 week follow up visit, at which time all outcome measures will be repeated. After completion of the 12 week follow-up period, participants will be invited to participate in qualitative focus groups to further explore their experience of this intervention. Participant flow through the study is outlined in Figure 1.

Sample size

The total number of participants recruited for this study will be 30. As this is a feasibility study, it has not been powered to detect a statistically significant difference in efficacy between the arms. In determining thresholds for progression criteria, based on previous trials of a similar nature, we expect at least 75% achievement[27 28]. If so, the 95% confidence interval will exclude 60% and therefore we have set this as the progression criteria for each criterion, requiring a sample size of 30 participants allocated in a 2:1 ratio, intervention:control.

Randomisation

All eligible, consenting participants will be randomised to one of the two trial arms (intervention or control), using permuted block design randomisation with blocks of 3 and 6. Allocation will be stratified by practice. An independent researcher will generate the set of sequences and assign participants to the intervention groups using sequentially numbered sealed envelopes to ensure allocation concealment until interventions are assigned. Due to the nature of this study, it will not be possible to further blind participants, clinicians or some of the study team to the treatment allocation beyond this point.

Intervention

The Diamond programme

The active intervention is a behaviourally informed, low-carbohydrate, low-energy diet delivered by healthcare professionals at a GP practice. It draws upon the motivational value of the relationship between the healthcare professional and the patient, but provides almost all the technical knowledge-transfer through the use of structured materials such as meal plans, thus addressing the uncertainties and lack of confidence expressed by health professionals about giving dietary advice[29].

For peer review only - http://bmjopen.bmj.com/site/about/guidelines.xhtml

The intervention aims to improve patients' adherence to the programme by providing a structured simple behavioural support programme, including goal-setting, planning, feedback and problem solving. It is designed to induce rapid changes in dietary composition and weight loss, followed by a sustainable maintenance programme. The intervention is envisaged to take 10-15 minutes of support from the GP and 80 minutes from the nurse to deliver over four sessions of support (baseline, week 2, week 4, and week 8).

This intervention was designed following the principles of the Person-Based Approach[30]. It involved examining recent systematic reviews, reviewing qualitative studies and process assessments of these types of interventions, and developing the behavioural and dietary intervention programme with a multi-disciplinary team comprising a diabetes-specialist dietician and clinician, primary care physicians, and patient involvement. Two groups of patients with type 2 diabetes, including those who had recently tried variations of a low-carbohydrate, low-energy diet, also met to discuss the intervention and to inform and advise on the dietary and behavioural support components.

The dietary component of this intervention consists of a low-carbohydrate and energy restricted diet (800-1000kcal/day) for 8 weeks, transitioning to a 4 week maintenance period. The core principles include: advice to exclude sugary and starchy foods high in carbohydrates (eg biscuits, confectionery, bread, pasta, potatoes) entirely from their diet (with the exception of dairy and limited fruit intake), strict portion control, and avoiding energy-dense foods. Standardised "healthy eating" advice regarding fresh vegetables, and lean meat and fish, are also included. After the initial 8 week intensive weight loss phase, participants will be advised to gradually increase their energy intake, re-introducing one 'normal-sized' meal at a time, until they remain weight stable, but with guidance on maintaining a sustainable lower-carbohydrate diet in the longer term.

The design of this intervention has been additionally informed by behavioural analysis, identifying domains of the Behaviour Change Wheel[31] and Theoretical Domains Framework[32] to promote successful behaviour change. Utilising these frameworks, the key influencing factors which have been targeted are: psychological capability (including knowledge, skills, decision processes and behavioural regulation), social and physical opportunity (social influences and environmental context), reflective motivation (beliefs about capabilities and consequences, optimism, intentions, goals and reinforcement).

To address these components, the intervention will include:

- A brief behavioural intervention consisting of both dietary and motivational advice, including the rationale behind the intervention, to be delivered by a healthcare

For peer review only - http://bmjopen.bmj.com/site/about/guidelines.xhtml

professional in primary care. The dietary advice includes the essential features of the diet – low energy and low carbohydrate content, as described above.

- Written resources, including advice for meal planning and food selection, and suggested recipes for "real food" choices – to empower patients with the knowledge and skills for decision-making about food.
- Personalised realistic goal setting (e.g. change in weight, HbA1c, number of diabetic medications)
- Diary to record self-assessment of programme adherence.
- Structured healthcare professional follow up, to provide support and contingency planning, and feedback on progress. Additionally, patients will be able to self-monitor their health with the use of home fingerprick-testing blood glucose metres, and BP machines, for additional motivation and monitoring of progress.
- Feedback from healthcare professional on personal changes in health parameters as a consequence of the intervention (changes in weight, BP, HbA1c).

Comparator

Participants randomised to the control arm will receive usual care at their week 0 study visit, comprising a single face-to-face appointment with a healthcare professional, during which they will receive standard dietary and lifestyle information based on the Diabetes UK "what is a healthy balanced diet for diabetes" leaflet. If their managing GP or nurse feels they warrant referral for further diabetic care or input (for example, diabetes education course), or intensification of medications, and would ordinarily have pursued this as part of their routine care outside of the trial, this will be permitted as forming part of "usual care", but will be documented.

Outcomes

Primary

The primary objective of this study is to test the feasibility of a low-carbohydrate, low-energy, behavioural intervention, comprising targeted advice from a health professional combined with written dietary information, to promote weight loss and improved glycaemic control in patients with type 2 diabetes. This feasibility study will then determine whether to progress to a full randomised control trial.

The following progression criteria will determine whether to progress to a full trial:

For peer review only - http://bmjopen.bmj.com/site/about/guidelines.xhtml

1. That 60% of allocated "intervention" group participants attempt the dietary intervention after randomisation.
   (Evaluated by documentation after intervention visit; assessed as the proportion of patients who agree to start the dietary intervention, as recorded by the nurse delivering the intervention session).
2. Fidelity of intervention delivery: That healthcare professionals conduct the intervention delivery session with at least 60% of essential elements present.
   (Evaluated by assessment of audio recorded consultations against a checklist of pre-specified essential elements).
3. That 60% of participants attend the final follow-up session
   (Documentation of final study visit)

Secondary

A mixture of quantitative and qualitative methods will be used to assess process measures and effectiveness measures. This feasibility study is not powered to detect statistical differences in efficacy outcomes but will examine the following parameters in order to test trial procedures, process, resources and management, to aid sample size estimates for a future trial, to determine the most appropriate primary outcome measures for a future trial, and to inform further development of the intervention strategy.

***Process Measures**:*

- Percentage of eligible patients, as a proportion of the total population of patiets in a practice, with type 2 diabetes.
- Percentage of people who fulfil the recruitment criteria who accept the invitation to participate
- Proportion of patients who enrol in the study who are deemed to have with "suboptimal control" (HbA1c above the NICE target of ≥7%)
- Participant adherence to the protocol: including, change in dietary composition (low-carbohydrate, energy restricted - assessed using 24 hour dietary recall questionnaires); participants' self-reported concordance with the intervention; availability of data for outcome measures; attendance at follow up sessions; contamination of the control group (i.e., those who choose to follow the principles of the intervention (i.e. follow a low-carbohydrate diet), despite being allocated to the control group). This will be assessed using 24 hour dietary recall questionnaires to establish change in dietary composition (frequency of carbohydrate consumption, energy restriction) of control group participants.

For peer review only - http://bmjopen.bmj.com/site/about/guidelines.xhtml

347 • Difference between "baseline" HbA1c value and that used from the latest record as inclusion criteria.

349 • Serious Adverse Events (SAEs) reported up to the end of the 12 week study participation period.

350 ***Exploratory outcomes***:

351 • Change in HbA1c - Number of patients previously in diabetic HbA1c range, now in "at risk of diabetes" (6-6.4%) or "diabetes in remission" (<6.0%, off medications) HbA1c range

353 • Change in fasting glucose, fasting insulin, HOMA-%B, HOMA-%S

354 • Change in weight

355 • Change in diabetic medication (number of diabetic medications currently prescribed to the patient; dose of diabetic medications; initiation of new medication during study period; initiation of insulin; initiation of injectable diabetic medication; number of medications stopped or changed during the study period)

359 • Change in lipid profile – total cholesterol, HDL, triglycerides, calculated non-HDL cholesterol, and total cholesterol:HDL ratio,

361 • Change in liver function tests (Bilirubin, ALT, AST, ALP, Albumin, AST:ALT)

362 • Change in blood pressure (systolic, diastolic)

363 • Change in antihypertensive medication (number of medications started and stopped during study period; dose of antihypertensive medications; initiation of new medication during study period)

366 • Change in medication prescribing costs (total and diabetic) across study group and total practice diabetic population

368 • Effect on patient's problem areas in diabetes (PAID) score

369 ***Qualitative Measures:***

370 • Experience of and acceptability of the intervention, for patients and healthcare practitioners (assessed using qualitative focus groups following completion of the initial study period).

372 Measurements

373 Figure 2 provides a summary of the measurements collected.

374 Sociodemographic characteristics

375 Participants will be asked to self-report age, sex and ethnicity, education history and employment status.

For peer review only

377 **Medical and medication history**

378 Relevant medical history and all current medication will be recorded and checked against the
379 participant's medical record.

380 **Physical measurements**

381 Height will be measured using stadiometers, to the nearest 1cm. Weight will be measured to the
382 nearest 0.1kg using a digital scale (SC-240 MA, Tanita Japan). BMI will be calculated using the
383 standard formula BMI=weight(kg)/height(m)^2. Blood pressure will be measured in triplicate after 5
384 minutes seated rest, with at least 1 minute between each measurement. All assessors will be
385 trained in standardised methods of taking physical measurements according to the study manual of
386 procedures.

387 **Fasting blood sample**

388 A fasting venous blood sample for HbA1c, fasting insulin, fasting glucose, liver function tests
389 (Bilirubin, ALT, AST, ALP, Albumin) and lipid profile (total cholesterol, triglycerides, HDL, calculated
390 LDL, and total:HDL cholesterol ratio) will be collected at baseline, 8 weeks and 12 weeks. All blood
391 samples will be taken, handled, analysed and disposed of according to standard NHS procedures and
392 local practice policy.

393 **Questionnaires**

394 During study follow-up visits, patients will be asked to complete questionnaires to assess the
395 following measures:

396 • *Quality of life:* the PAID score (Problem Areas in Diabetes), a 20 item questionnaire measuring
397 problems related to emotions, treatment, food and social support[33], will be measured at
398 baseline and 12 weeks.

399 • *Dietary composition:* a 24 hour dietary recall questionnaire will be used to assess intake of
400 different food groups at baseline, 2, 8 and 12 weeks.

401 • *Motivation:* Self-reported motivation and perceptions across domains of diet, health and
402 diabetes control will be assessed using a 6 point questionnaire at baseline and 12 weeks.

403 • *Self-reported adherence to intervention:* Self-reported adherence to the 3 core components of
404 the dietary intervention will be assessed at 2, 4, 8 and 12 weeks.

For peer review only - http://bmjopen.bmj.com/site/about/guidelines.xhtml

Retention and withdrawal

Each participant will have the right to withdraw from the study at any time. In addition, the investigator may discontinue a participant from the study at any time if they consider it necessary for any reason, including ineligibility (either arising during the study or having been overlooked at screening). If a participant requests to withdraw from the study, it will be explained to them that we would like to use their data collected up to the point at which they have withdrawn from the study, unless they request that we do not do so. The reason for withdrawal will be recorded in the CRF. Withdrawn participants will not be replaced in this feasibility study. To reflect the burden of participation and promote participant retention and complete follow up, participants will be offered a £20 gift card upon attending the 12 week follow-up appointment.

Adverse events

We will record and report all serious adverse events (SAEs) following Good Clinical Practice and standard HRA processes. The duration of the SAE recording period for each participant lasts from their enrolment on to the study, to their completion of the study. We elected not to record other AEs because this process is burdensome for clinicians and participants and we considered that this programme was unlikely to induce significant AEs.

Data management

Data will be recorded on hard copy CRFs and subsequently entered into a web-based data capture system (RedCap, © 2018 Vanderbilt University) with data stored on a secure server hosted by the Primary Care Clinical Trials Unit at the University of Oxford. The system has an inbuilt audit trail facility and ability to run internal validation checks.

Statistical analysis

Primary outcome measures for this study are progression criteria, to inform any future RCT powered to detect an intervention effect. Analysis for progression criteria will utilise data from all participants recruited to the trial. Descriptive statistical methods along with inferential statistics presenting confidence intervals will be used to analyse and report progression criteria.

Secondary outcome measures for this study include process measures, and exploratory effectiveness measures. Both measures will use all collected data, including that from participants who have not

For peer review only - http://bmjopen.bmj.com/site/about/guidelines.xhtml

completed the trial, and from participating practices (where pertaining to proportions of patients identified as eligible, and responding to invitations to participate). Descriptive comparative summary statics (e.g. difference in means or proportions, together with 95% confidence intervals, comparison to baseline and overall change compared to control group) will be used.

Acceptability and experience of the intervention by participants and healthcare professionals will be collected as qualitative data from interviews and focus groups conducted after intervention completion. These data will be qualitatively analysed using thematic analysis, and reported descriptively.

Patient involvement

This study evolved through patient demand in the local area, to know more about dietary options and in particular low-carbohydrate diets for people with type 2 diabetes. We convened two panels of patients with type 2 diabetes, one consisting of members who had recently tried variations of a low-carbohydrate, low-energy diet, to inform and advise on the dietary and behavioural support components of the intervention, the patient materials, and the perceived benefits or burdens of the study for patients. One patient member has subsequently joined the trial management group and will be involved in trial steering decisions during the monitoring and running of the trial, and regarding progressing to a full-scale trial.

Qualitative substudy

The purpose of the nested qualitative sub-study is to explore patients' and healthcare professionals' experience of receiving, enacting and delivering this intervention, and of participating in the study. We will seek the views of up to 10 patient participants from the intervention group, from those who consent to be contacted for the nested qualitative study, and 6-8 healthcare professionals. The patient participants chosen will aim to reflect a range of success with weight loss and glycaemic control, with thought given to not mixing those who have found the programme easy or successful, with those who have not achieved their goals. Additionally, we will aim to capture a breadth of participant characteristics (age, gender, social class, ethnicity, or, for the healthcare professional interviews, the participant's profession). All healthcare professionals who have been involved in the study will be invited to take part. Should more participants volunteer than are required, purposive sampling will be used to ensure both GPs and nurses are represented in the cohort, and to include those who have conducted a greater number of sessions.

For peer review only - http://bmjopen.bmj.com/site/about/guidelines.xhtml

Focus groups will be conducted after all participants have completed their 3 month study follow up period. Written informed consent will be taken before the focus group commences. All focus groups will be audio recorded, and these audio recordings will be transcribed. Patients will be reimbursed for their time and practices will be reimbursed for the healthcare professionals taking time out from their clinical duties to participate.

Focus groups will follow broad topic areas based upon the study objectives, but will encourage participants to discuss their perceptions and experiences freely and in depth. Topics will include: the acceptability of the intervention; views on which components of their treatment group they felt were effective and which were not effective; thoughts about impact on experience of diabetes and its management; thoughts about weight management going forwards. Data will be analysed using thematic analysis.

ETHICS AND DISSEMINATION

The study protocol (V2.0 1.3.18) was reviewed and approved by the South Central Oxford B REC Committee (Ref: 18/SC/0071) and registered on the ISRCTN (62452621). Any protocol modifications will be submitted for review by the research ethics committee and amended at the trial registry.

If the trial proves feasible, according to the specified progression criteria it is planned that the results will inform design of a full-scale randomised trial to test the efficacy of the intervention to improve glycaemic control.

The findings of this feasibility study will be submitted for publication in a peer-reviewed journal, and presented at conferences, to disseminate the results to academic and health professional audiences, and made available to participants and to a wider public on our website at the time of publication.

AUTHOR CONTRIBUTIONS:

EM, PA and SJ developed the concept for the study and wrote the first draft of the protocol. EM, PA, SJ and PD prepared the study documents and coordinated the HRA and ethics application. EM drafted the manuscript for publication, with input from PA and SJ. PD, MN, CB, RF, KH and GT were involved in the detailed design of the study, and approved the final protocol and manuscript.

ACKNOWLEDGEMENTS: The authors would like to thank the members of the DIAMOND patient participation panels for their invaluable input and advice throughout all stages of study design.

For peer review only

For peer review only - http://bmjopen.bmj.com/site/about/guidelines.xhtml

FUNDING STATEMENT: PA and SAJ are NIHR Senior Investigators and funded by NIHR CLAHRC Oxford. PA, SAJ, GDT, PD, and MN are supported by the NIHR Oxford BRC. This work was supported by the National Institute for Health Research (NIHR) Oxford Biomedical Research Centre (BRC) and the School for Primary Care Research (SPCR) (grant reference number 404).

DISCLAIMER: This report is independent research by the National Institute for Health Research. The views expressed in this publication are those of the author(s) and not necessarily those of the NHS, the University of Oxford, the NIHR or the Department of Health.

COMPETING INTERESTS: PA and SAJ have received research grant funding but no personal renumeration from commercial weight loss companies, but none of these companies have interests in the programme described here. CB is the author of "The 8-week blood sugar diet recipe book", published in 2016. PAD is a member of the PHE/SACNE/DUK committee reviewing evidence on the effects of low carbohydrate diets in people with type 2 diabetes.

DATA STATEMENT: For access to the study data set, a formal request should be sent to the DIAMOND study group. The request will only be considered when the principal results of the study have been published.

CHECKLIST FOR APPROPRIATE REPORTING STATEMENT: SPIRIT 2013 Checklist

FIGURE LEGENDS:

FIGURE 1: Participant flow through the study

FIGURE 2: Schedule of measurements

REFERENCES:

1. DiabetesUK. Facts and Stats. *Diabetes UK website, wwwdiabetesorguk* 2015
2. Mokdad AH, Ford ES, Bowman BA, et al. Diabetes trends in the U.S.: 1990-1998. *Diabetes care* 2000;23(9):1278-83. doi: 10.2337/diacare.23.9.1278
3. Nield L, Moore H, Hooper L, et al. Dietary advice for treatment of type 2 diabetes mellitus in adults. *Cochrane Database of Systematic Reviews* 2007(3) doi: 10.1002/14651858.CD004097.pub4
4. NICE_Guidance. NG28 Type 2 diabetes in adults: Management. 2015
5. Look_Ahead_Research_Group. Reduction in Weight and Cardiovascular Disease Risk Factors in Individuals With Type 2 Diabetes: One-Year Results of the Look AHEAD Trial. *Diabetes care* 2007;30(6):1374-83. doi: 10.2337/dc07-0048
6. Wing RR. Long-term effects of a lifestyle intervention on weight and cardiovascular risk factors in individuals with type 2 diabetes mellitus: four-year results of the Look AHEAD trial. *Archives of internal medicine* 2010;170(17):1566-75. doi: 10.1001/archinternmed.2010.334 [published Online First: 2010/09/30]

For peer review only - http://bmjopen.bmj.com/site/about/guidelines.xhtml

7. Stratton IM, Adler AI, Neil HAW, et al. Association of glycaemia with macrovascular and microvascular complications of type 2 diabetes (UKPDS 35): prospective observational study. *BMJ* 2000;321(7258):405-12. doi: 10.1136/bmj.321.7258.405

8. Wing RR, Lang W, Wadden TA, et al. Benefits of modest weight loss in improving cardiovascular risk factors in overweight and obese individuals with type 2 diabetes. *Diabetes care* 2011;34(7):1481-6. doi: 10.2337/dc10-2415 [published Online First: 2011/05/20]

9. Lean MEJ, Leslie WS, Barnes AC, et al. Primary care-led weight management for remission of type 2 diabetes (DiRECT): an open-label, cluster-randomised trial. *The Lancet* 2018;391(10120):541-51. doi: 10.1016/S0140-6736(17)33102-1

10. Evert AB, Boucher JL, Cypress M, et al. Nutrition Therapy Recommendations for the Management of Adults With Diabetes. *Diabetes care* 2014;37(Supplement 1):S120-S43. doi: 10.2337/dc14-S120

11. Ajala O, English P, Pinkney J. Systematic review and meta-analysis of different dietary approaches to the management of type 2 diabetes. *The American journal of clinical nutrition* 2013;97(3):505-16. doi: 10.3945/ajcn.112.042457 [published Online First: 2013/02/01]

12. DiabetesUK. Evidence-Based Guidelines for the Prevention and Treatment of Diabetes. *wwwdiabetesorguk* 2011

13. Arora SK, McFarlane SI. The case for low carbohydrate diets in diabetes management. *Nutrition & Metabolism* 2005;2:16-16. doi: 10.1186/1743-7075-2-16

14. Sainsbury E, Kizirian NV, Partridge SR, et al. Effect of dietary carbohydrate restriction on glycemic control in adults with diabetes: A systematic review and meta-analysis. *Diabetes research and clinical practice* 2018;139:239-52. doi: https://doi.org/10.1016/j.diabres.2018.02.026

15. Dyson P. Low Carbohydrate Diets and Type 2 Diabetes: What is the Latest Evidence? *Diabetes Therapy* 2015;6(4):411-24. doi: 10.1007/s13300-015-0136-9

16. van Wyk HJ, Davis RE, Davies JS. A critical review of low-carbohydrate diets in people with Type 2 diabetes. *Diabetic medicine : a journal of the British Diabetic Association* 2016;33(2):148-57. doi: 10.1111/dme.12964 [published Online First: 2015/09/29]

17. Snorgaard O, Poulsen GM, Andersen HK, et al. Systematic review and meta-analysis of dietary carbohydrate restriction in patients with type 2 diabetes. *BMJ Open Diabetes Research & Care* 2017;5(1) doi: 10.1136/bmjdrc-2016-000354

18. Meng Y, Bai H, Wang S, et al. Efficacy of low carbohydrate diet for type 2 diabetes mellitus management: A systematic review and meta-analysis of randomized controlled trials. *Diabetes research and clinical practice* 2017;131:124-31. doi: 10.1016/j.diabres.2017.07.006 [published Online First: 2017/07/28]

19. Huntriss R, Campbell M, Bedwell C. The interpretation and effect of a low-carbohydrate diet in the management of type 2 diabetes: a systematic review and meta-analysis of randomised controlled trials. *European journal of clinical nutrition* 2018;72(3):311-25. doi: 10.1038/s41430-017-0019-4 [published Online First: 2017/12/23]

20. Fan Y DH, Chen G, Mao X, Liu C. Effects of low carbohydrate diets in individuals with type 2 diabetes: systematic review and meta-analysis. *Int J Clin Exp Med* 2016;9(6):11166-74.

21. Castaneda-Gonzalez LM, Bacardi Gascon M, Jimenez Cruz A. Effects of low carbohydrate diets on weight and glycemic control among type 2 diabetes individuals: a systemic review of RCT greater than 12 weeks. *Nutricion hospitalaria* 2011;26(6):1270-6. doi: 10.1590/s0212-16112011000600013 [published Online First: 2012/03/14]

22. Dyson P. Low Carbohydrate Diets and Type 2 Diabetes: What is the Latest Evidence? *Additional data via personal communication. Diabetes Therapy* 2015;6(4):411-24. doi: 10.1007/s13300-015-0136-9

23. Whitington AaI. "Fixing Dad". *Whitington Brothers Films; Broadcast BBC 2 24072016* 2016

24. Rangan Chatterjee RP, Tim Harcourt, Carrie Britton, Jack Rampling. Doctor in the House. *Studio Lambert Production; Broadcast BBC2 6116* 2015

For peer review only

For peer review only - http://bmjopen.bmj.com/site/about/guidelines.xhtml

25. Unwin M, Unwin, J. Low carbohydrate diet to achieve weight loss and improve HbA1c in type 2 diabetes and pre-diabetes: experience from one general practice,. *Practical Diabetes* 2014;31(2)

26. JLA. James Lind Alliance Priority Setting partnerships: Diabetes (Type 2) Top 10. http://wwwjlanihracuk/priority-setting-partnerships/diabetes-type-2/top-10-uncertaintieshtm *(accessed July 2018)* 2017

27. Leslie WS, Ford I, Sattar N, et al. The Diabetes Remission Clinical Trial (DiRECT): protocol for a cluster randomised trial. *BMC family practice* 2016;17(1):1-10. doi: 10.1186/s12875-016-0406-2

28. Aveyard P, Lewis A, Tearne S, et al. Screening and brief intervention for obesity in primary care: a parallel, two-arm, randomised trial. *Lancet* 2016 doi: 10.1016/s0140-6736(16)31893-1 [published Online First: 2017/01/04]

29. Huang J, Yu H, Marin E, et al. Physicians' weight loss counseling in two public hospital primary care clinics. *Academic medicine : journal of the Association of American Medical Colleges* 2004;79(2):156-61. [published Online First: 2004/01/28]

30. Yardley L, Morrison L, Bradbury K, et al. The Person-Based Approach to Intervention Development: Application to Digital Health-Related Behavior Change Interventions. *J Med Internet Res* 2015;17(1):e30. doi: 10.2196/jmir.4055

31. Michie S, van Stralen MM, West R. The behaviour change wheel: a new method for characterising and designing behaviour change interventions. *Implementation science : IS* 2011;6:42. doi: 10.1186/1748-5908-6-42 [published Online First: 2011/04/26]

32. Michie S, Johnston M, Abraham C, et al. Making psychological theory useful for implementing evidence based practice: a consensus approach. *Quality & safety in health care* 2005;14(1):26-33. doi: 10.1136/qshc.2004.011155 [published Online First: 2005/02/05]

33. Polonsky WH, Anderson BJ, Lohrer PA, et al. Assessment of diabetes-related distress. *Diabetes care* 1995;18(6):754-60. [published Online First: 1995/06/01]

For peer review only

For peer review only - http://bmjopen.bmj.com/site/about/guidelines.xhtml

Figure 1: Participant flow through the study

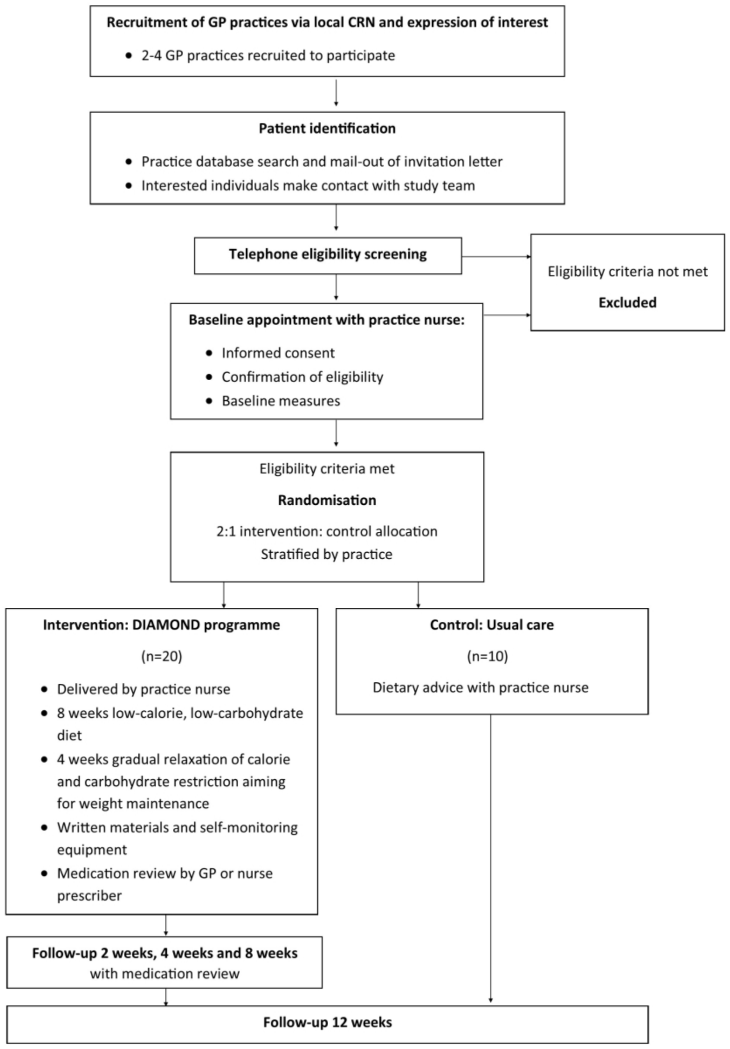

Figure 1: Participant flow through the study

209x297mm (300 x 300 DPI)

Figure 2: Schedule of measurements

	STUDY PERIOD					
	Enrolment	Allocation	Study Visits			Follow up
**TIMEPOINT**	*Baseline*	0	*Week 2*	*Week 4*	*Week 8*	*Week 12*
**ENROLMENT:**						
Eligibility screen	X					
Informed consent	X					
Randomisation	X					
**INTERVENTION GROUPS:**						
DIAMOND programme		X	X	X	X	X
Usual Care		X	X			X
**ASSESSMENTS:**						
Demographics	X					
Medical history	X					X
Medication review	X		X	X	X	X
Weight	X		X	X	X	X
Height	X		X	X	X	X
Blood pressure	X		X	X	X	X
Fasting blood sample	X				X	X
Adverse event assessment			X	X	X	X
24 hour dietary recall questionnaire	X		X		X	X
Motivation assessment	X					X
Problem Areas in Diabetes (PAID) score	X					X
Self-reported adherence to intervention	X		X	X	X	X

Figure 2: Schedule of measurements

209x297mm (300 x 300 DPI)

STANDARD PROTOCOL ITEMS: RECOMMENDATIONS FOR INTERVENTIONAL TRIALS

SPIRIT 2013 Checklist: Recommended items to address in a clinical trial protocol and related documents*

Section/item	Item No	Description	Addressed on page number
**Administrative information**			
Title	1	Descriptive title identifying the study design, population, interventions, and, if applicable, trial acronym	1____________
Trial registration	2a	Trial identifier and registry name. If not yet registered, name of intended registry	1_____________
	2b	All items from the World Health Organization Trial Registration Data Set	Y_____________
Protocol version	3	Date and version identifier	_16____________
Funding	4	Sources and types of financial, material, and other support	17_____________
Roles and responsibilities	5a	Names, affiliations, and roles of protocol contributors	1,16,17_________
	5b	Name and contact information for the trial sponsor	1
	5c	Role of study sponsor and funders, if any, in study design; collection, management, analysis, and interpretation of data; writing of the report; and the decision to submit the report for publication, including whether they will have ultimate authority over any of these activities	17
	5d	Composition, roles, and responsibilities of the coordinating centre, steering committee, endpoint adjudication committee, data management team, and other individuals or groups overseeing the trial, if applicable (see Item 21a for data monitoring committee)	N/A___________

For peer review only

For peer review only - http://bmjopen.bmj.com/site/about/guidelines.xhtml

Introduction

Background and rationale	6a	Description of research question and justification for undertaking the trial, including summary of relevant studies (published and unpublished) examining benefits and harms for each intervention	3-5____________
	6b	Explanation for choice of comparators	3-5_____________
Objectives	7	Specific objectives or hypotheses	5______________
Trial design	8	Description of trial design including type of trial (eg, parallel group, crossover, factorial, single group), allocation ratio, and framework (eg, superiority, equivalence, noninferiority, exploratory)	5______________

Methods: Participants, interventions, and outcomes

Study setting	9	Description of study settings (eg, community clinic, academic hospital) and list of countries where data will be collected. Reference to where list of study sites can be obtained	5______________
Eligibility criteria	10	Inclusion and exclusion criteria for participants. If applicable, eligibility criteria for study centres and individuals who will perform the interventions (eg, surgeons, psychotherapists)	6-7____________
Interventions	11a	Interventions for each group with sufficient detail to allow replication, including how and when they will be administered	8-9_____________
	11b	Criteria for discontinuing or modifying allocated interventions for a given trial participant (eg, drug dose change in response to harms, participant request, or improving/worsening disease)	14______________
	11c	Strategies to improve adherence to intervention protocols, and any procedures for monitoring adherence (eg, drug tablet return, laboratory tests)	8,14__________
	11d	Relevant concomitant care and interventions that are permitted or prohibited during the trial	9_____________
Outcomes	12	Primary, secondary, and other outcomes, including the specific measurement variable (eg, systolic blood pressure), analysis metric (eg, change from baseline, final value, time to event), method of aggregation (eg, median, proportion), and time point for each outcome. Explanation of the clinical relevance of chosen efficacy and harm outcomes is strongly recommended	10-12 ______________
Participant timeline	13	Time schedule of enrolment, interventions (including any run-ins and washouts), assessments, and visits for participants. A schematic diagram is highly recommended (see Figure)	20______________

For peer review only - http://bmjopen.bmj.com/site/about/guidelines.xhtml

Sample size	14	Estimated number of participants needed to achieve study objectives and how it was determined, including clinical and statistical assumptions supporting any sample size calculations	8_____________
Recruitment	15	Strategies for achieving adequate participant enrolment to reach target sample size	14_____________
**Methods: Assignment of interventions (for controlled trials)**			
Allocation:			
Sequence generation	16a	Method of generating the allocation sequence (eg, computer-generated random numbers), and list of any factors for stratification. To reduce predictability of a random sequence, details of any planned restriction (eg, blocking) should be provided in a separate document that is unavailable to those who enrol participants or assign interventions	8_____________
Allocation concealment mechanism	16b	Mechanism of implementing the allocation sequence (eg, central telephone; sequentially numbered, opaque, sealed envelopes), describing any steps to conceal the sequence until interventions are assigned	_8____________
Implementation	16c	Who will generate the allocation sequence, who will enrol participants, and who will assign participants to interventions	_8____________
Blinding (masking)	17a	Who will be blinded after assignment to interventions (eg, trial participants, care providers, outcome assessors, data analysts), and how	8_____________
	17b	If blinded, circumstances under which unblinding is permissible, and procedure for revealing a participant's allocated intervention during the trial	_8____________
**Methods: Data collection, management, and analysis**			
Data collection methods	18a	Plans for assessment and collection of outcome, baseline, and other trial data, including any related processes to promote data quality (eg, duplicate measurements, training of assessors) and a description of study instruments (eg, questionnaires, laboratory tests) along with their reliability and validity, if known. Reference to where data collection forms can be found, if not in the protocol	10-14_________
	18b	Plans to promote participant retention and complete follow-up, including list of any outcome data to be collected for participants who discontinue or deviate from intervention protocols	_14____________

For peer review only - http://bmjopen.bmj.com/site/about/guidelines.xhtml

Data management	19	Plans for data entry, coding, security, and storage, including any related processes to promote data quality (eg, double data entry; range checks for data values). Reference to where details of data management procedures can be found, if not in the protocol	14_____________
Statistical methods	20a	Statistical methods for analysing primary and secondary outcomes. Reference to where other details of the statistical analysis plan can be found, if not in the protocol	_14-15_________
	20b	Methods for any additional analyses (eg, subgroup and adjusted analyses)	15_____________
	20c	Definition of analysis population relating to protocol non-adherence (eg, as randomised analysis), and any statistical methods to handle missing data (eg, multiple imputation)	_____________
**Methods: Monitoring**			
Data monitoring	21a	Composition of data monitoring committee (DMC); summary of its role and reporting structure; statement of whether it is independent from the sponsor and competing interests; and reference to where further details about its charter can be found, if not in the protocol. Alternatively, an explanation of why a DMC is not needed	n/a___________
	21b	Description of any interim analyses and stopping guidelines, including who will have access to these interim results and make the final decision to terminate the trial	14___________
Harms	22	Plans for collecting, assessing, reporting, and managing solicited and spontaneously reported adverse events and other unintended effects of trial interventions or trial conduct	14_____________
Auditing	23	Frequency and procedures for auditing trial conduct, if any, and whether the process will be independent from investigators and the sponsor	14_____________
**Ethics and dissemination**			
Research ethics approval	24	Plans for seeking research ethics committee/institutional review board (REC/IRB) approval	16_____________
Protocol amendments	25	Plans for communicating important protocol modifications (eg, changes to eligibility criteria, outcomes, analyses) to relevant parties (eg, investigators, REC/IRBs, trial participants, trial registries, journals, regulators)	16_____________

For peer review only - http://bmjopen.bmj.com/site/about/guidelines.xhtml

Consent or assent	26a	Who will obtain informed consent or assent from potential trial participants or authorised surrogates, and how (see Item 32)	_7_____________
	26b	Additional consent provisions for collection and use of participant data and biological specimens in ancillary studies, if applicable	_7_____________
Confidentiality	27	How personal information about potential and enrolled participants will be collected, shared, and maintained in order to protect confidentiality before, during, and after the trial	_7_____________
Declaration of interests	28	Financial and other competing interests for principal investigators for the overall trial and each study site	_17_____________
Access to data	29	Statement of who will have access to the final trial dataset, and disclosure of contractual agreements that limit such access for investigators	17_____________
Ancillary and post-trial care	30	Provisions, if any, for ancillary and post-trial care, and for compensation to those who suffer harm from trial participation	_n/a___________
Dissemination policy	31a	Plans for investigators and sponsor to communicate trial results to participants, healthcare professionals, the public, and other relevant groups (eg, via publication, reporting in results databases, or other data sharing arrangements), including any publication restrictions	16_____________
	31b	Authorship eligibility guidelines and any intended use of professional writers	_____________
	31c	Plans, if any, for granting public access to the full protocol, participant-level dataset, and statistical code	17_____________
**Appendices**			
Informed consent materials	32	Model consent form and other related documentation given to participants and authorised surrogates	_____________
Biological specimens	33	Plans for collection, laboratory evaluation, and storage of biological specimens for genetic or molecular analysis in the current trial and for future use in ancillary studies, if applicable	_____________

*It is strongly recommended that this checklist be read in conjunction with the SPIRIT 2013 Explanation & Elaboration for important clarification on the items. Amendments to the protocol should be tracked and dated. The SPIRIT checklist is copyrighted by the SPIRIT Group under the Creative Commons "Attribution-NonCommercial-NoDerivs 3.0 Unported" license.

For peer review only - http://bmjopen.bmj.com/site/about/guidelines.xhtml
